# VEGFR2 promotes central endothelial activation and the spread of pain in inflammatory arthritis

**DOI:** 10.1016/j.bbi.2018.03.012

**Published:** 2018-11

**Authors:** Nicholas Beazley-Long, Catherine Elizabeth Moss, William Robert Ashby, Samuel Marcus Bestall, Fatimah Almahasneh, Alexandra Margaret Durrant, Andrew Vaughan Benest, Zoe Blackley, Kurt Ballmer-Hofer, Masanori Hirashima, Richard Phillip Hulse, David Owen Bates, Lucy Frances Donaldson

**Affiliations:** a Arthritis Research UK Pain Centre & School of Life Sciences, Medical School, University of Nottingham, Nottingham NG7 2UH, UK; b Cancer Biology, School of Medicine, QMC, University of Nottingham, Nottingham NG7 2UH, UK; c Paul Scherer Institute, Villingen, Switzerland; d Department of Physiology and Cell Biology, Kobe University Graduate School of Medicine, Japan; e COMPARE University of Birmingham and University of Nottingham Midlands, UK

**Keywords:** Inflammatory pain, Rheumatoid arthritis, Chronic pain, Mechanical allodynia, VEGFR2, Glio-vascular activation, ICAM-1, CD11b, Microglia, mono-arthritis

## Abstract

Chronic pain can develop in response to conditions such as inflammatory arthritis. The central mechanisms underlying the development and maintenance of chronic pain in humans are not well elucidated although there is evidence for a role of microglia and astrocytes. However in pre-clinical models of pain, including models of inflammatory arthritis, there is a wealth of evidence indicating roles for pathological glial reactivity within the CNS. In the spinal dorsal horn of rats with painful inflammatory arthritis we found both a significant increase in CD11b^+^ microglia-like cells and GFAP^+^ astrocytes associated with blood vessels, and the number of activated blood vessels expressing the adhesion molecule ICAM-1, indicating potential glio-vascular activation. Using pharmacological interventions targeting VEGFR2 in arthritic rats, to inhibit endothelial cell activation, the number of dorsal horn ICAM-1^+^ blood vessels, CD11b^+^ microglia and the development of secondary mechanical allodynia, an indicator of central sensitization, were all prevented. Targeting endothelial VEGFR2 by inducible Tie2-specific VEGFR2 knock-out also prevented secondary allodynia in mice and glio-vascular activation in the dorsal horn in response to inflammatory arthritis. Inhibition of VEGFR2 *in vitro* significantly blocked ICAM-1-dependent monocyte adhesion to brain microvascular endothelial cells, when stimulated with inflammatory mediators TNF-α and VEGF-A_165_a. Taken together our findings suggest that a novel VEGFR2-mediated spinal cord glio-vascular mechanism may promote peripheral CD11b^+^ circulating cell transmigration into the CNS parenchyma and contribute to the development of chronic pain in inflammatory arthritis. We hypothesise that preventing this glio-vascular activation and circulating cell translocation into the spinal cord could be a new therapeutic strategy for pain caused by rheumatoid arthritis.

## Highlights

• Targeting VEGFR2 prevents secondary allodynia in inflammatory arthritis models.
• Anti-VEGFR2 reduces vascular ICAM-1 and microglia in the dorsal horn.
• Anti-VEGFR2 inhibits monocyte attachment to brain endothelial cells.
• We propose a novel glio-vascular-immune mechanism that promotes pain.
• Therapeutic anti-VEGFR2 may lessen chronic pain in inflammatory arthritis.

## 1 Introduction

Pain experienced by rheumatoid arthritis patients is chronic, debilitating, and can persist despite adequate control of inflammation (Taylor et al., 2010, Heiberg and Kvien, 2002, McWilliams and Walsh, 2016). The mechanisms that drive pain in rheumatoid arthritis are complex and involve peripheral inflammation, joint damage, peripheral nociceptive processes and central sensitization, the latter being thought to underpin chronic pain. Because of this complex aetiology, patients can experience unprovoked pain in varied forms (Walsh and McWilliams, 2012), and suffer from allodynia (pain to innocuous stimuli) and hyperalgesia (heightened sensation of painful stimuli) (Meeus et al., 2012, Wendler et al., 2001). In addition, pain-associated psychological distress and fatigue further disrupt quality of life (Roche et al., 2003, Ulus et al., 2011). Current therapies for rheumatoid arthritis aim to control systemic and joint-associated inflammation and have a degree of success (Walsh and McWilliams, 2012), but often fail to control pain. There still remains, therefore, a pressing need for better control for the pain that persists in the face of optimal inflammatory control (McWilliams and Walsh, 2016, Emery, 2012).

Chronic inflammatory diseases such as rheumatoid arthritis are also associated with widespread endothelial ‘dysfunction’ increasing cardiovascular risk in these patients (Steyers and Miller, 2014). The central nervous system (CNS) microvasculature, including the spinal cord, is unlike microvasculature elsewhere in the mammalian body, forming a more highly selective blood-brain/spinal cord barrier (BBB/BSCB) which maintains neuronal homeostasis. The BBB/BSCB contains endothelial cells, vascular pericytes (Graeber et al., 1990), astrocytic end-feet, and perivascular macrophages (Hickey and Kimura, 1988, Graeber et al., 1989). Astrocytic end-feet ensheathe the microvasculature and regulate vessel function. For example, release of astrocytic endothelin-1 is vasoconstrictive (Macrae et al., 1993) and astrocytic monocyte attractant protein-1 promotes leukocyte migration across the human brain endothelium, which can be blocked by inhibiting intercellular adhesion molecule-1 (ICAM-1) (Weiss et al., 1998). More recent research indicates microglial tumor necrosis factor-alpha (TNF-α) stimulates glio-vascular activation, promoting endothelial prostaglandin I2 receptor expression and this mechanism contributes to the generation of neuropathic pain (Kanda et al., 2017). In contrast to the CNS microvasculature the microvascular barrier in sensory dorsal root ganglia (DRG) is fenestrated and less restrictive (Abram et al., 2006, Anzil et al., 1976, Jacobs et al., 1976, Segond von Banchet et al., 2009).

Immune cells (monocytes/macrophages, lymphocytes, neutrophils and mast cells), glia (astrocytes and microglia) and vascular endothelia can all contribute to the development of sensitized pain pathways in both the CNS and the periphery [reviewed by (Scholz and Woolf, 2007)]. Microglia are CNS innate immune cells derived from proliferating residential microglia or circulating myeloid cells that transmigrate into the parenchyma (Hess et al., 2004, Eglitis and Mezey, 1997, Lawson et al., 1992, Yao et al., 2016). Stimulated microglia can release pro-nociceptive factors such as TNF-α, interleukin-1β and interleukin-6 (Ledeboer et al., 2005, Raghavendra et al., 2004, Ohtori et al., 1976). These promote increased firing of neurons, enhancing information transfer (Konig et al., 2016, Reeve et al., 2000), and in nociceptive systems potentially contributing to a sensitized neuronal state (central sensitization). Consequently, agents that inhibit microglial activity, such as minocycline, have anti-nociceptive properties in preclinical pain models (Ledeboer et al., 2005, Zhang et al., 2012, Chen et al., 2013, Sagar et al., 2011).

Along with TNF-α, vascular endothelial growth factor-A (VEGF-A) protein is increased in the serum of rheumatoid arthritis patients (Nakahara et al., 2003). VEGF-A and TNF-α are co-related (Nowak et al., 2008), with TNF-α driving endothelial expression of both VEGF-A_165_a and endothelial adhesion molecules (Haraldsen et al., 1996, Radisavljevic et al., 2000). Anti-TNF-α therapy (e.g. etanercept) can significantly reduce VEGF-A levels in rheumatoid arthritis (Strunk et al., 2006, Macias et al., 2005). VEGF-A levels in patients that do not respond to anti-TNF therapy can remain significantly elevated or even increase further (Knudsen et al., 2009) indicating TNF-independent VEGF-A expression in non-responders and suggests an alternative therapeutic target. VEGF-A regulates blood vessel function including endothelial activation through VEGF receptor-2 (VEGFR2): the pro-angiogenic VEGF-A splice variant VEGF-A_165_a triggers endothelial expression of chemokines and cell surface adhesion molecules including ICAM-1 (Kim et al., 2001), and stimulates immune cell transmigration across brain endothelial cells (Lee et al., 2002). In addition recombinant human (rh)VEGF-A_165_a (full receptor agonist) has pro-nociceptive effects whereas the partial VEGF receptor agoinst rhVEGF-A_165_b has opposing actions on nociception (Hulse et al., 2014, Hulse et al., 2015). Targeting specifically VEGFR2 in normal animals including by intrathecal inhibitor injection also significantly affects rodent nociceptive behavior (Hulse et al., 2014, Hulse et al., 2016).

To date it is unclear how targeting VEGFR2 disrupts nociceptive processes. With the knowledge that anti-VEGFR2 is anti-nociceptive in preclinical models of neuropathic pain, we hypothesized that targeting VEGFR2 would be anti-nociceptive in an inflammatory mono-arthritis model. As leukocytes transmigrate into the spinal cord parenchyma and dorsal root ganglia in other preclinical pain models (Zhang et al., 2016, Zhang et al., 2007), and VEGF-A is an endothelial ‘activator’ we also hypothesized that spinal dorsal horn and dorsal root ganglion endothelia would be activated in inflammatory arthritis. As a consequence to endothelial activation, we hypothesised that targeting VEGFR2 would reduce endothelial activation and the number of reactive spinal cord microglia thereby providing a novel mechanism for the anti-nociceptive effect of anti-VEGFR2 *in vivo*, and a novel target for the treatment of chronic pain in inflammatory arthritis.

## 2 Materials and methods

All animal procedures were performed in accordance with the United Kingdom Animals (Scientific Procedures) Act 1986/Amendment Regulations 2012 and with University of Nottingham Animal Welfare and Ethical Review Group approval. Animals were maintained under 12 h/12 h light/dark-cycle, 21 ± 2 °C with standard chow and water *ad libitum*. A total of 70 male Wistar rats (Charles River UK, 200–250 g starting weight) and 119 transgenic experimental mice were used in this study.

### 2.1 Animal housing design, group randomization and sample size

Experimental group allocation was randomized per cage while ensuring that in each cage there was a mix of all groups to minimise possible inter-cage interactions, with the exception of vehicle-dosed *vegfr2^fl/fl^* Tie2CreER^T2^ mice: tamoxifen used for Tie2CreER^T2^ induction (see below) and its active metabolites are present in urine (Kisanga et al., 2005). This excretion could lead to cross-contamination between vehicle- and tamoxifen-dosed animals in the same cage so these mice were housed separately. Rats (two-tier cages) and mice (single-tier cages) were housed four and four-to-six per cage in individually ventilated cages respectivefully. Animal welfare checks were performed daily throughout the experiments. The primary measure was ipsilateral mechanical threshold behavior therefore sample sizes were based on previous experiments using these models (Chillingworth and Donaldson, 2003, Hsieh et al., 2015, Kelly et al., 2007) and an unpublished pilot study.

### 2.2 Induction of articular inflammation

Complete Freund’s adjuvant (CFA) was prepared by resuspending heat-denatured *mycobacterium tuberculosis* [UK Ministry of Agriculture Fisheries and Food, now known as DEFRA, UK] in mineral oil at 2 mg/mL, sonicated (1 h) and filtered (40 μm pore size). Rats underwent unilateral intra-articular (tibiofemoral) injection of 100 μg CFA (in 50 μL oil) under brief isoflurane anesthesia (2–3% in oxygen). Non-injected control rats were age/sex-matched and used to control for the inflammation. CFA vehicle was not injected into control animals because the oil and joint damage from the injection are sufficient to cause inflammation confounding the interpretation of results. Transgenic mice underwent unilateral sub-cutaneous, *peri*-articular (tibiotarsal) injection of CFA [2 × 80 μg CFA in 40 μL oil, on either side of the joint (Chillingworth and Donaldson, 2003) under isoflurane anesthesia (2–3% in oxygen) two weeks after tamoxifen induction. Sham mice underwent anesthesia and injection site preparation without CFA injection. Thus this was a non-inflamed control group matching the rat study i.e. a control group for total inflammation and not the action of CFA alone.

### 2.3 Systemic antibody and pharmacological inhibitor administration

VEGFR2 signaling was targeted by i.p. injection of: 20 μg/kg VEGF-A_165_b in PBS [VEGF receptor competitive antagonist (Kawamura et al., 2008), anti-angiogenic VEGF-A isoform (Woolard et al., 2004), received from K. Ballmer-Hoffer]; 15 mg/kg PTK787 in PBS [VEGFR1/VEGFR2 inhibitor, vatalanib dihydrochloride, Selleckchem (Wood et al., 2000) with high BBB penetration (Remko et al., 2011), or 12 μg/kg DC101 in PBS [neutralizing murine-specific anti-VEGFR2 antibody (IgG mAb, BioXcell)]. To control for off-target effects of DC101 immunoglobulin (IgG), control animals were given species/concentration-matched IgG (MP Biomedicals, same route/vehicle). Intra-peritoneal inhibitor injection was performed immediately after intra-articular CFA and at later time points (see results).

### 2.4 Local antibody and pharmacological inhibitor administration

VEGFR2 signaling was targeted in the right tibiofemoral joint by intra-articular (*local*) injection on days 0 and 7 of VEGF-A_165_b [20 ng injected with CFA (day 0) and in 50 μL PBS (day 7)] or PTK787 [100 pmol injected with CFA (day 0) and in 50 μL PBS (day 7)].

### 2.5 Measurement of nociceptive behaviors and joint swelling

All behavioral assays were conducted blinded to the experimental groups. Animals were habituated to operator handling and testing environments over 3 days prior to testing. Animals were allowed 20 min to re-acclimatize prior to testing. Mechanical stimulus-withdrawals were recorded as previously described (Hulse et al., 2014): von Frey monofilaments (mouse: 0.04–2 g; rat: 0.16–26 g) were applied five times to each hind paw, for ∼5 s and the percentage of response scored. The 50% withdrawal threshold was calculated from the stimulus–response relationship [generated by plotting percentage of response against the logarithm (base 10) of applied force] using Graphpad Prism 7. Ipsilateral and contralateral (to CFA injection) hind limb weight distribution was measured using an incapacitance tester (Linton Instrumentation, UK). The weight borne by each hind limb was averaged over 3 s and the mean of three readings calculated. Results are displayed as ipsilateral hind limb weight bearing as a percentage of total weight borne on the hind limbs. Tibiofemoral joint diameter in awake rats and tibiotarsal joint diameter in awake mice were recorded using a digital caliper. Readings were taken in triplicate and mean values calculated.

### 2.6 Spinal cord and DRG immunofluorescence

Eight and 11 days after induction of articular inflammation rats were terminally anesthetized with sodium pentobarbital (60 mg/ml) and perfuse-fixed with ∼250 mL ice cold PBS + heparin (1U/mL) followed by ∼250 mL ice cold 4% paraformaldehyde (PFA) in PBS. Tissue was collected at these time points as significant secondary allodynia had developed in these rats and changes in pain behavior with treatment were observed on the preceding days (days 7 and 10). The study design did not permit the full suite of behaviour measurements and tissue collection on the same day. In our experience of this model secondary mechanical allodynia peaks after one week (Drake et al., 2016) and is maintained to at least day 14 therefore we selected time points for tissue analysis when secondary pain was established. Mouse spinal cords were perfuse-fixed as above and dissected following the completion of the behavioral tests on day 14, a time point at which significant differences in contralateral behavior were observed. Spinal columns were placed into PFA overnight, then into 30% sucrose in PBS for upto 72 h. Spinal cord lumbar enlargement (central site corresponding to hind paw sensory input and site of secondary sensitivity, L4-L5 (Butler and Comparative, 2005)and ipsi- and contralateral lumbar dorsal root ganglia (ganglia corresponding to the majority of sensory input from knee, L3 (Russell et al., 2012)were dissected and frozen in optimal cutting temperature (OCT) compound (VWR, UK) on dry ice. Spinal cord (20 μm) and DRG (20 μm) cryosections were cut and thaw-mounted onto slides (SuperFrost® Plus, VWR). Sections were rehydrated in PBS + 0.2% Triton X-100 (PBS-X) and blocked (10% fetal bovine serum, 5% bovine serum albumin in PBS-X) for 2 h at room temperature. Due to the thickness of the sections, primary antibodies in blocking solution were incubated overnight at 4 °C and slides were washed with PBS-X (×3, 10 min). Slides were incubated overnight with secondary antibodies in blocking solution (4°C, humid conditions) and washed as above. Finally slides were incubated with streptavidin for 2 h at room temperature, washed as above and mounted in Fluoroshield. Primary antibodies: glial fibrillary acid protein (GFAP) (Abcam, ab7260, pAb anti-serum) used at 1 in 500; CD11b (microglial/monocyte marker) (BioRad, MCA275) used at 0.2 μg/mL; ICAM-1 (activated endothelial cell marker) (Abcam, ab171123) used at 0.2 μg/mL; IB4 (Sigma, L3759) used at 1 μg/mL; VEGFR2 (Cell Signaling, 55B11) used at 1 in 250, CD31 used at 5 μg/mL (R&D Systems, AF3628). Secondary antibodies (goat anti-mouse/rabbit, Alexa Fluor-conjugated, Life Technologies) all used at 1 in 1000; streptavidin-488 (Life Technologies) used at 1 in 500, Hoechst543334 used at 1 μg/mL.

### 2.7 Confocal microscopy and analysis

**Fig. 1:**
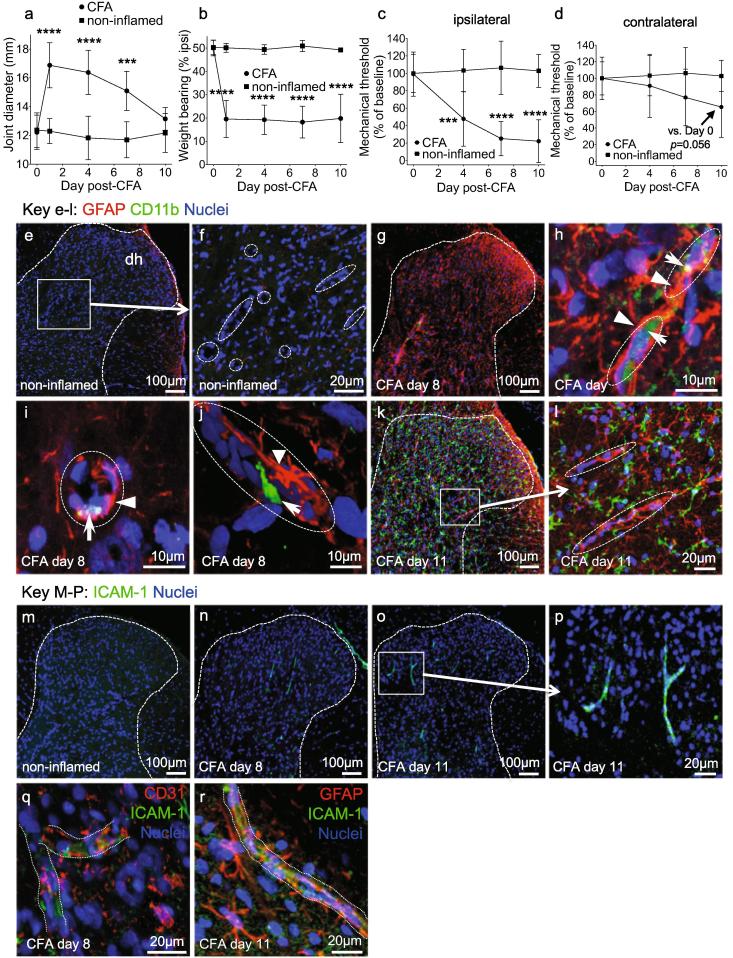
Articular inflammation drives secondary mechanical allodynia, glio-vascular activation and microglia reactivity in the spinal cord. Tibiofemoral joint diameter (a), weight borne on the ipsilateral hind paw (b), ipsilateral mechanical stimulus withdrawal threshold (c) and contralateral mechanical stimulus (d) withdrawal threshold after intra-articular CFA (single dose, 100 μg), n = 9 (CFA), n = 4 (non-inflamed controls). Immunoreactivity of GFAP and CD11b antibodies in the dorsal horn 8 and 11 days following induction of inflammatory arthritis (e-l). Representative images (e,g,k) showing the increase in GFAP (red) and CD11b (green) immunoreactivity in the dorsal horn in (e-f) non-inflamed control, (g-j) day eight and (k-l) day 11 following induction of articular inflammation with CFA (nuclei- blue). High magnification imaging revealed microvessels, identified by strings of flat (longitudinal) or curved (cross-sectional) nuclei (blue), surrounded by GFAP^+^ astrocytic end-feet (red, arrowhead) and associated with CD11b^+^ cells (green, arrows) at day eight (h,i). 2D image following z-stack reconstruction showing a CD11b^+^ cell with a cellular projection within the vessel lumen (j). Wide spread dorsal horn CD11b^+^ microglia-like staining within the dorsal horn parenchyma by day 11 (k-l). Representative images showing ICAM-1 expression in the dorsal horn of non-inflamed controls (m-r) and CFA-treated animals on day 8 (n) and 11 (m). ICAM-1^+^ microvessel fragments (p) were observed throughout the dorsal horn. ICAM-1^+^ immunoreactivity was associated with CD31^+^ vessels (q) and vessels wrapped in GFAP^+^ astrocytic end-feet (r). Statistical analysis: two-way repeated measures analysis of variance + Dunnett’s multiple comparisons test. CFA + vehicle vs. respective baseline: ***p,<0.001, ****p < 0.0001. Group size = 8–9. Data displayed as mean ± SD. (For interpretation of the references to colour in this figure legend, the reader is referred to the web version of this article.)

Fluorescence images were captured using Leica SPE confocal microscope using ×10, ×40 and ×63 oil immersion objective lenses and Leica Applications Suite software (LAS X). ImageJ software was used for image analysis. CD11b^+^/Hoechst total dorsal horn cell counts were automated using ImageJ: the dorsal horn (laminae I-V) was traced, then CD11b^+^ and Hoechst 1024x1024 resolution 8-bit images were thresholded with consistent settings across all groups, and the percentage of overlay determined using the particle analyzer and measure functions. Nuclei overlayed by >35% of the CD11b^+^ signal were deemed CD11b^+^ cells. This percentage was optimized to match manual counts from pilot manual analysis. Dorsal horn GFAP, CD11b or ICAM-1 immunofluorescent signal was manually quantified as being vessel-associated if the signal localized with a vessel. In the rat study a GFAP, CD11b or ICAM-1-associated vessel was identified if the fluorescent signal localised with nuclear staining indicative of vessels: longitudinal/oblique vessels were identified by a chain of two or more elongated flat nuclei (endothelial cells or pericytes, dotted elipses, Fig. 1h,i,l) and cross-sectional dorsal horn vessels were identified by two or more curved nuclei forming a crescent or complete ring (endothelial cells or pericytes dotted elipses, Fig. 1f,i). In the Tie2-CreER^T2^ VEGFR2 KO study the vessel localization was aided with CD31 co-staining. All manual ICAM-1^+^, CD11b^+^ and GFAP^+^ vessel analyses were performed with the analyser blinded to experimental group. The number of ICAM-1^+^, CD11b^+^ or GFAP^+^-associated vessel fragments per animal were counted and expressed over the total sectional area per animal analysed to account for differences in the total area analysed.

### 2.8 Generation of Tie2-CreER^T2^ x *vegfr2*^fl/fl^ mice

To elucidate the site of action of the anti-VEGFR2 agents we crossed mice that were homozygous for the VEGFR2 gene with floxP sites either side of exon 1 (*vegfr2^fl/fl^)* (Albuquerque et al., 2009) with Tie2CreER^T2^ mice [European Mutant Mouse Archive EM:00715 (Forde et al., 2002) to generate inducible endothelial *vegfr2* knock-out adult mice. Experiments were conducted on F3/F4 mice of both sexes. All mice used were confirmed homozygous for the VEGFR2-loxP transgene (*vegfr2^fl/fl^*) and either heterozygous or wild-type for Tie2CreER^T2^.

### 2.9 Tamoxifen Tie2CreER^T2^*vegfr2*^fl/fl^ knock-out

Tamoxifen (Sigma, T5648) was dissolved in ethanol then diluted 1 in 10 in autoclaved sunflower oil (vehicle 10% ethanol, 90% oil) (Acharya et al., 2011). Five daily doses of tamoxifen (1 mg/100 μL i.p.) or vehicle (volume/route-matched) were administered to either Tie2CreER^T2^-positive or Tie2CreER^T2^-negative mice as a control for actions of tamoxifen.

### 2.10 CD31^+^ cell selection for endothelial cell knock-out characterisation

Two to four weeks after tamoxifen/vehicle induction, mice were transcardially perfused (20 mL PBS + heparin 10U/mL), ∼100 mg lung or spinal cord tissue dissected, washed twice with PBS and enzymatically (0.125% collagenase P, 1 h, 37 °C) and mechanically dissociated (triturated with P1000 micropipette), cell dispersate passed through a 40 μm cell filter and entered into a mouse CD31^+^ selection microbead assay (20 million cells) using MS columns and following the manufacturer’s instructions (Miltenyibiotech).

### 2.11 VEGFR2 genomic DNA and mRNA characterisation

Following CD31-positive cell selection genomic DNA (Qiagen, DNeasy kit) and RNA (Chomczynski and Sacchi, 1987) (CD31-positive, column flow-through and input fractions) were extracted and purified. Genomic DNA (∼100 ng) was amplified by conventional PCR with 1 forward primer (F) and 2 reverse primers (R1, R2) designed to detect wild-type *vegfr2* gene (F-R1, 322 bp) and/or loxP-flanked *vegfr2* gene (F1-R3, not knocked out 439 bp, or knock-out product 218 bp) as performed previously (Albuquerque et al., 2009, Sison et al., 2010) with annealing temperature 65 °C, primers: F; 5 -CTTTCCACTCCTGCCTACCTAG-3; R1, 5–TGGAGAGCAAGGCGC-TGCTAGC-3; R2, 5 -AATTTGGGTGCCATAGCCAATC-3). To investigate changes in VEGFR2 mRNA by RT-PCR 500 μg RNA was treated with DNaseI and reversed transcribed with 500 ng Oligo-d(T) and 250 ng random primers and M-MLV reverse transcriptase (all Promega). 1 μL of the total 20 μL complementary DNA (cDNA) was added to 2.5 μL Taqman probe +9 μL ddH_2_O +12.5 μL ddPCR Supermix for probes (Bio-Rad). Droplets were generated by adding the 25 μL to 70 μL Droplet oil (Bio-Rad) using a QX100 droplet generator (Bio-Rad). Amplification of VEGFR2 or CD31 cDNA was carried out using standard Taqman protocols (10 min activation step 95 °C, 40 cycles of 30 s denaturation at 94 °C, 1 min annealing at 60 °C and final 10 min extension at 98 °C using a PCR thermal cycle (Bio-Rad) with VEGFR2 (KDR, Mm01222421_m1) and PECAM1 (CD31, mM01242584_mi) Taqman Primers (Life Technologies). Samples were analysed using a QX100 droplet reader (Bio-Rad). Thresholding was manually performed, based on negative and positive control results only when automatic thresholding of values was not possible.

### 2.12 VEGFR2/Tie2 flow cytometry

Following CD31^+^ lung or spinal cord cell selection, cells were incubated with calceinAM (2.5 μM, 20′, 37 °C), then stained with fluorophore-labeled antibodies against mouse Tie2 (PE-conjugated, Biolegend #124008, 10 μg/mL) and mouse VEGFR2 (APC-conjugated, Biolegend #136406, 10 μg/mL), and total nuclei stained with Hoechst543334. Flow cytometry was performed (MoFlo Astrio Cell Sorter) to determine changes in proportions of living Tie2^+^ and VEGFR2^+^ cells. Single dye control groups identified that no signal compensations were required between the four dyes (Hoechst543334: 350–488/59 nm, Calcein: 488–513/26 nm, PE: 561–579/16 nm, APC: 640–671/30 nm). In samples from lung, side and forward scatter of calcein-positive (living) cells revealed two distinct populations, a low Tie2/VEGFR2-negative population and a high Tie2/VEGFR2 population. Changes in Tie2 and VEGFR2 expression in these two populations were investigated between tamoxifen-induced and uninduced (vehicle-treated) mice. In samples from spinal cord, distinct populations of living cells were not evident when analysed by side and forward scatter properties so all living CD31^+^ cells were analysed for Tie2 and VEGFR2 expression.

### 2.13 Human brain endothelial cell – Monocyte adhesion assay

Human brain microvascular endothelial cells (HBMEC) were cultured in RPMI + 20% FBS, 2 mM L-glutamine, 1 mM sodium pyruvate, 1% MEM non-essential amino acids, 1% MEM vitamin, 1% penicillin/streptomycin. Human monocytes (THP-1, Sigma) were cultured in RPMI + 10% FBS, 1% penicillin/streptomycin. HBMEC were plated in 96 well plates at 30,000 cells/well and treatments were performed in RPMI + 1% FBS, 1% penicillin/streptomycin. Endothelial monolayers were pretreated overnight with either VEGF-A_165_b, ZM323881 (20 nM) or PTK787 (100 nM) prior to 24 h treatment with recombinant human TNF-α (R&D Systems). A blocking ICAM-1 antibody (100 ng/mL) or a concentration and species-matched IgG (100 ng/mL, ms IgG) was added with TNF-α treatment and again at the time of monocyte addition. Monocytes were fluorescently tagged with 2.5 μM calcein-AM for 1 h at 37 °C, washed in warm PBS, added to HMBEC monolayer at 30,000 cells per well and incubated for 2 h for attachment. Unattached monocytes were washed off and cells fixed with 4% PFA. Calcein fluorescence was measured on fluorescence plate reader (VictorX4, Perkin-Elmer). Results shown have had the mean fluorescence value of the untreated group (without stimulation) subtracted and then are expressed as a percentage of the mean fluorescence of the maximal response.

### 2.14 Western blotting

HBMEC and human umbilical vein microvascular cell (HUVEC) protein lysate was prepared from ∼ 80% confluent cultured cells in RIPA lysis buffer with protease inhibitor cocktail (P8340, Sigma). Samples (10 μg) were separated by SDS-PAGE on a 4–20% gradient gel (Bio-Rad Mini-PROTEAN® TGX™ Precast, 90 V for 90 min) and transferred onto nitrocellulose membrane by turbo transfer (Bio-Rad TransBlot Turbo, 25 V for 7 min). Membranes were incubated with either α-occludin (71–1500, rabbit, 0.5 µg/mL), α-von Willebrand Factor (ab6994, rabbit, 17 μg/mL) or α-actin (sc1615, goat, 0.5ug/ml was used as a loading control). Secondary antibodies used were IRDye® 680RD donkey anti-rabbit IgG, IRDye® 800CW donkey anti-mouse or IRDye® 800CW donkey anti-Goat IgG, all at 0.2 µg/mL and membranes visualized on LiCor Odyssey Fc.

### 2.15 Data extraction and statistical analysis

Acquired data were processed and graphed using GraphPad Prism v6. All data were Gaussian distributed and are shown as mean ± standard deviation. For statistical comparisons each animal was an experimental unit. Three or more groups were compared using one- or two-way analysis of variance with *post-hoc* Dunnett’s multiple comparisons tests to test for significant differences to the respective control group. Spearman correlation analysis was performed to test for significant correlation between mechanical threshold scores and histological data.

## 3 Results

### 3.1 Intra-articular complete Freund’s adjuvant drives dorsal horn astrocytic, microglial and microvascular reactivity

We first investigated pain behavior and the reactivity of dorsal horn microglia, astrocytes and microvessels in response to intra-articular CFA. CFA caused a significant increase in tibiofemoral diameter Fig. 1a), a significant decrease in weight borne on the ipsilateral hind paw Fig. 1b) and a significant decrease in the ipsilateral hind paw mechanical threshold Fig. 1c). The contralateral hind paw mechanical threshold decreased by day 10 to 65% of baseline indicating sensiziation but this did not reach statistical significance compared with respective baseline (*p* = 0.056) nor compared with non-inflamed controls (*p* = 0.071), when analysed by 2-way ANOVA + post hoc analysis Fig. 1d). The significant decrease in ipsilateral hind paw mechanical threshold (secondary mechanical allodynia) indicated the development of central sensitization. In many chronic painful diseases and animal models reactive astrocytes and microglia in the dorsal horn contribute to central sensitization and the generation of pain (Taylor et al., 2017, Mifflin and Kerr, 2017), therefore we investigated changes in dorsal horn glial fibrillary acidic protein (GFAP, reactive astrocytes) and CD11b (reactive microglia) expression at the spinal level corresponding to sensory input from the site of secondary sensitivity (sensitized hind paw, L5). Eight days after intra-articular CFA, GFAP expression was more prominent (red, denoted by arrowheads, Fig. 1 h,i,j) than in non-inflamed controls Fig. 1e-f), and there was a small number of CD11b^+^ cells (green, denoted by arrows, Fig. 1 g-j), which were not observed in non-inflamed controls. At this time point a proportion of GFAP immunoreactivity and the majority of observed CD11b^+^ cells were associated with nuclear staining indicative of microvessels Fig. 1 h-j), and image reconstruction demonstrated that the GFAP^+^ processes (reactive astrocytic end-feet) were wrapped round vessel-like structures in which CD11b^+^ cells were present (highlighted in Fig. 1i,j). For illustrative purposes nuclear staining indicative of microvessels is encircled by dashed ellipses in high magnification images Fig. 1f,h,i,j,l). At day 11 GFAP imunoreactivity was associated with microvessels while CD11b^+^ cells were widespread throughout the dorsal horn parenchyma Fig. 1 k,l). The number of GFAP^+^ reactive astrocytic end-feet associated with microvessel nuclear staining in the ipsilateral dorsal horn increased from 0.41 ± 0.07 × 10^−5^ μm^−2^ (non-inflamed) to 1.35 ± 0.09 × 10^−5^ μm^−2^ (day 8) and 1.02 ± 0.22 × 10^−5^ μm^−2^ (day 11). At day 11, CD11b^+^ microglia-like cells were widespread in the dorsal horn parenchyma Fig. 1 k,l) and had increased from 0.05% ± 0.09 (non-inflamed) to 8.93% ± 1.17 (% of total cells).

CD11b is a natural ligand for ICAM-1 (Springer, 1990), a key molecule in the adhesion and extravasation of leukocytes through activated vascular endothelium following spinal cord injury (Isaksson et al., 1999). Increased ICAM-1 expression in dorsal horn-activated endothelium as a result of peripheral inflammation could contribute to transendothelial migration of CD11b^+^ cells into the CNS parenchyma. Compared with non-inflamed animals Fig. 1m) there was indeed a significant increase in the number of ICAM-1^+^ vessels throughout the dorsal horn of CFA animals at days eight and 11 (green elongate structures associated with microvessel nuclear staining, Fig. 1n-p), (non-inflamed 0.22 ± 0.22 × 10^-5^ μm^-2^, day 8 11.67 ± 0.10 × 10^-5^ μm^-2^, day 11 11.64 ± 02.07 × 10^-5^ μm^-2^). ICAM-1 and CD31 co-staining confirmed ICAM-1^+^ microvessels in the dorsal horn of CFA-treated animals Fig. 1q). GFAP (red) and ICAM-1 (green) co-staining also identified ICAM-1^+^ microvessels associated with GFAP^+^ astrocytic end-feet Fig. 1r) in the dorsal horn of CFA animals.

### 3.2 Intra-peritoneal but not intra-articular delivery of anti-VEGFR2 agents is anti-nociceptive in inflammatory arthritis

**Fig. 2:**
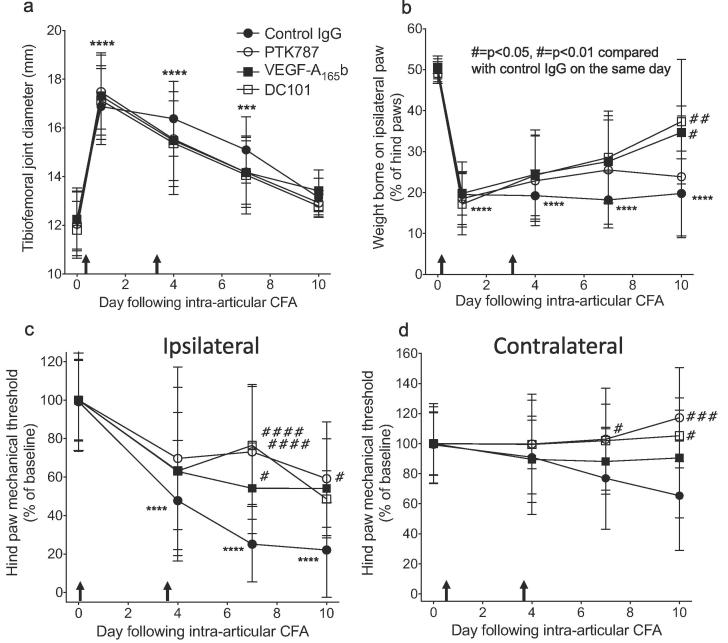
Systemic delivery of anti-VEGFR2 agents prevents the development of secondary mechanical allodynia. Tibiofmoral joint diameter after intra-articular CFA + two i.p. injections (arrows) of either a murine VEGFR2-specific neutralising antibody (DC101), VEGF-A_165_b or PTK787 compared with control (Control IgG) (a). Weight bearing on the paw ipsilateral to CFA injection after treatment with either DC101 (murine neutralizing VEGFR2 mAb), PTK787 (VEGFR1&2 inhibitor) or VEGF-A_165_b (competitive VEGF receptor antagonist) (b). Ipsilateral hind paw mechanical sensitivity after CFA and VEGFR2 inhibition (c). Contralateral hind paw sensitivity (d). Two statistical analyses were performed: two-way repeated measures analysis of variance + Dunnett’s multiple comparisons tests: CFA + Control IgG vs. respective baseline, *p < 0.05, **p,<0.01, ***p < 0.0001; or CFA + drug vs. CFA + Control IgG at given time point, ♯p < 0.05; ♯♯p < 0.01, ♯♯♯p < 0.0001, ♯♯♯♯p < 0.0001. Group size = 5–9. Data displayed as mean ± SD.

**Fig. 3:**
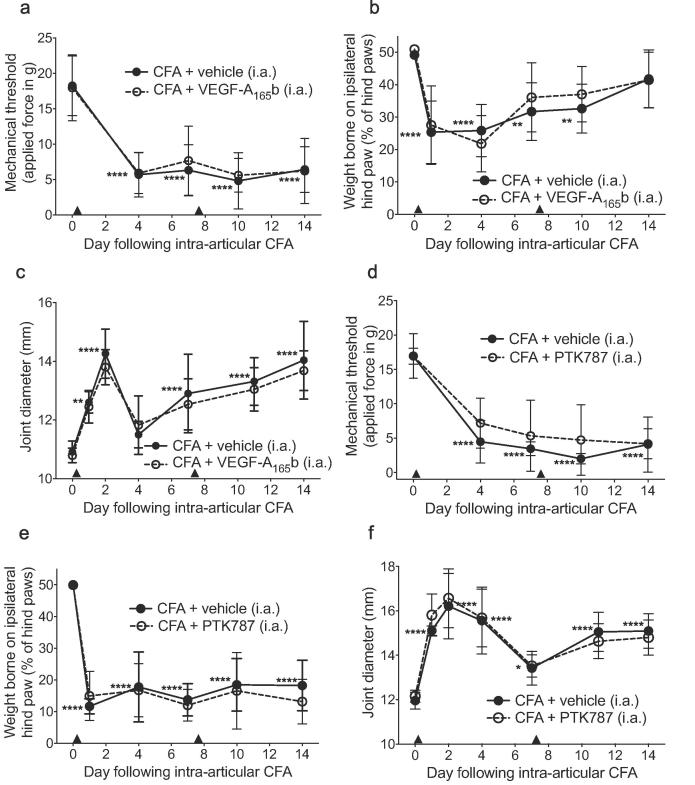
Intra-articular (tibiofemoral) delivery of VEGF receptor inhibitors did not affect the development of pain-associated behaviors in the CFA model of inflammatory monoarthritis. Intra-articular (i.a) CFA (single dose) plus two i.a. vehicle injections (arrow heads) caused a significant reduction in ipsilateral mechanical stimulus withdrawal threshold (a,d) at all time points measured, weight borne on ipsilateral hind paw (b,e) (days 1–10) and a significant increase in tibiofemoral joint diameter (c,f) compared to respective baseline (day 0). CFA + two i.a. injections of VEGF-A_165_b or PTK787 (arrow heads) had no effect on the development of the pain-associated behaviors and joint diameter (a-f). Two statistical analyses were performed: 2-way ANOVA + Dunnett’s multiple comparisons tests: CFA + vehicle vs. respective baseline, *p < 0.05, **p < 0.01, ***p < 0.0001; or CFA + vehicle vs. CFA + drug, #*p* < 0.05, ##*p* < 0.01, ###*p* < 0.0001. A-C n = 10, D-F n = 5. Data displayed as mean ± SD.

The spinal endothelial activation suggested that reducing central endothelial activation could be a route to preventing vascular-associated detrimental changes within the CNS. As VEGF-A has been shown to induce ICAM-1 through VEGFR2 (Usui et al., 2004) and we have previously found that VEGF-A is involved in pain states (Hulse et al., 2014, Hulse et al., 2015, Hulse et al., 2016), we sought to investigate whether inhibiting VEGFR2 by systemic delivery of anti-VEGFR2 agents affects the development of pain-associated behaviors through central rather than peripheral actions. To target VEGFR2 we used a naturally occurring VEGFR2 partial agonist, VEGF-A_165_b (Kawamura et al., 2008) that inhibits vascular action (Ourradi et al., 2017) and has anti-nociceptive properties (Hulse et al., 2014, Hulse et al., 2015), a small molecule VEGFR1&2 inhibitor with high BBB penetration, PTK787 (Remko et al., 2011) that also has anti-nociceptive properties (Hulse et al., 2014, Hulse et al., 2015) and a murine-specific VEGFR2 neutralising antibody that would not freely cross the BBB, DC101. Two systemic doses (days 0 and 3) of either VEGF-A_165_b (20 μg/kg i.p., Fig. 2), PTK787 (15 mg/kg i.p., Fig. 2) or DC101 (12 mg/kg i.p., Fig. 2) did not significantly affect the joint diameter compared to control Fig. 2a). The VEGFR2 neutralising antibody DC101 and VEGF-A_165_b both had a significant inhibitory effect on the shift in weight bearing by day 10 Fig. 2b). All three anti-VEGFR2 treatments significantly inhibited the development of secondary mechanical allodynia in the ipsilateral hind paw compared CFA + control on day 7 Fig. 2c). CFA induced a drop in contralateral hind paw threshold in control animals although this did not reach significance from respective baseline. However on day 10 both VEGF-A_165_b and DC101 treatments caused a signficant increase in weight borne on the ipsilateral paw compared to day 10 control animals Fig. 2d). In contrast to systemic delivery, intra-articular anti-VEGF agents (VEGF-A_165_b or PTK787) did not affect the development of secondary mechanical allodynia in the ipsilateral hind paw Fig. 3a,d), the shift in hind paw weight bearing to the contralateral side Fig. 3b,e), nor joint swelling Fig. 3c,e). These results indicate that *systemic* inhibition of VEGFR2 signalling, but not *local* inhibition in the knee, is anti-nociceptive, suggesting a mechanism that is distinct from the inhibition of local VEGF-A receptors in the joint. Furthermore, the effects on mechanical allodynia of all three systemically administered anti-VEGF agents, including a specific VEGFR2-neutralizing IgG DC101, suggest a central site of action. The anti-nociceptive effect of DC101, which would not freely cross into the CNS parenchyma, indicates a possible site of inhibition within the vasculature itself.

### 3.3 The effect of anti-VEGFR2 agents on glia reactivity in the dorsal horn of the spinal cord

**Fig. 4:**
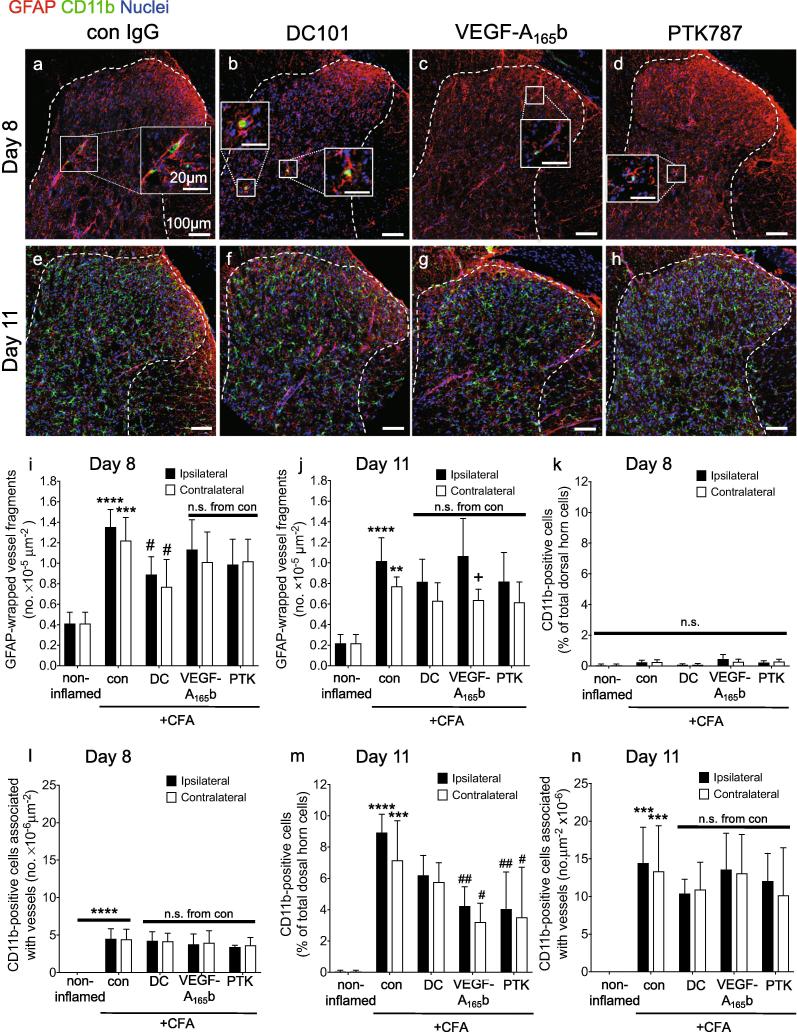
The effect of anti-VEGFR2 agents on astrocytic and microglial reactivity to articular inflammation. Intra-articular CFA caused a significant increase in the number of GFAP^+^ end-feet-wrapped vessel fragments in the ipsilateral dorsal horn on both (a) day eight and (e) day 11. Treatment with DC101 (b,f), but not VEGF-A_165_b (c,g) nor PTK787 (d,h) significantly reduced the number of ipsilateral and contralateral GFAP^+^ end-feet-wrapped vessel fragments on day eight (i), but not on day 11 (j). Intra-articular CFA caused a significant increase in microvessel-associated CD11b^+^ cells (ipsi- and contralateral) on day eight (k) and day 11 (l) with no difference observed between the CFA groups at both time points. On day eight there was no significant difference in the number of CD11b^+^ cells in the dorsal horn parenchyma compared to control (m), whereas on day 11 there was a significant increase in CD11b^+^ cells in the dorsal horn parenchyma compared to non-inflamed control animals. All three anti-VEGF treatments reduced this number although only VEGF-A_165_b (ipsi & contalateral) and PTK787 (ipsilateral) reached statistical significance (n). The only significant difference between respective ipsi- and contralateral effects was observed in VEGF-A_165_b group on the number of GFAP-wrapped vessels at day 11 (j). Three statistical analyses were performed: 2-way analysis of variance + Dunnett’s multiple comparisons test: control vs. non-inflamed *p < 0.5, **p < 0.01, ***p < 0.001, ****p < 0.0001; anti-VEGFR2 agent vs. control (ipsi or contra): #p < 0.5, ##p < 0.01, ###p < 0.001, ####p < 0.0001 and ipsi vs. contra of respective group +p < 0.5. Day eight: n = 4; day 11: n = 4–5. Data displayed as mean ± SD.

As anti-VEGFR2 agents supressed secondary pain behavior when administered systemically rather than locally, we investigated changes in CFA-induced reactive glia and microvessels in the dorsal horn after anti-VEGFR2 treatment. The number of GFAP-wrapped vessel-like structures was enhanced in both ipsilateral and contralateral dorsal horn in the CFA + control group at both day eight Fig. 4a-d,i) and day 11 Fig. 4e-h,j), and only DC101 treatment significantly affected the number of GFAP-wrapped vessels (ipsilateral, day 8). As observed in Fig. 1(g-j), a small, non-significant number of CD11b^+^ cells were detected in the dorsal horn parenchyma after 8 days compared with non-inflamed control animals Fig. 4a-d,k; Fig. 1i,j). However, of those that were detected, a significant number (CD11b^+^ cells, arrows Fig. 4a-d) were detected in association with the dorsal horn microvasculature of all CFA groups, suggesting an upregulation of CD11b^+^ cells in the microcirculation associated with peripheral inflammation Fig. 4a-d,l). By eleven days after intra-articular CFA there was a significantly increased number of CD11b^+^ cells within both the ipsi- and contralateral dorsal horn parenchyma of CFA-treated animals Fig. 4e-h,m). All VEGF inhibitory treatments reduced the number of CD11b^+^ cells in the spinal cord parenchyma on day 11, but this reached significance only for VEGF-A_165_b and PTK787. A significant number of these CD11b^+^ cells were associated with the microvasculature compared to non-inflamed control and compared to the number on each respective day eight with no observed difference between all CFA groups Fig. 4e-h,n). This suggested that the treatments had reduced the migration of CD11b^+^ cells from blood vessels into the surrounding parenchyma. The significant increase in dorsal horn CD11b^+^ cell number on day 11 significantly and negatively correlated with ipsilateral hind paw mechanical threshold (Supplementary Fig. S1). No differences were observed between ipsi- and contralateral sides within each group except in the VEGF-A_165_b group (number of GFAP-wrapped vessels, day 11, Fig. 4j).

### 3.4 The effect of anti-VEGFR2 agents on dorsal horn vascular ICAM-1 expression

**Fig. 5:**
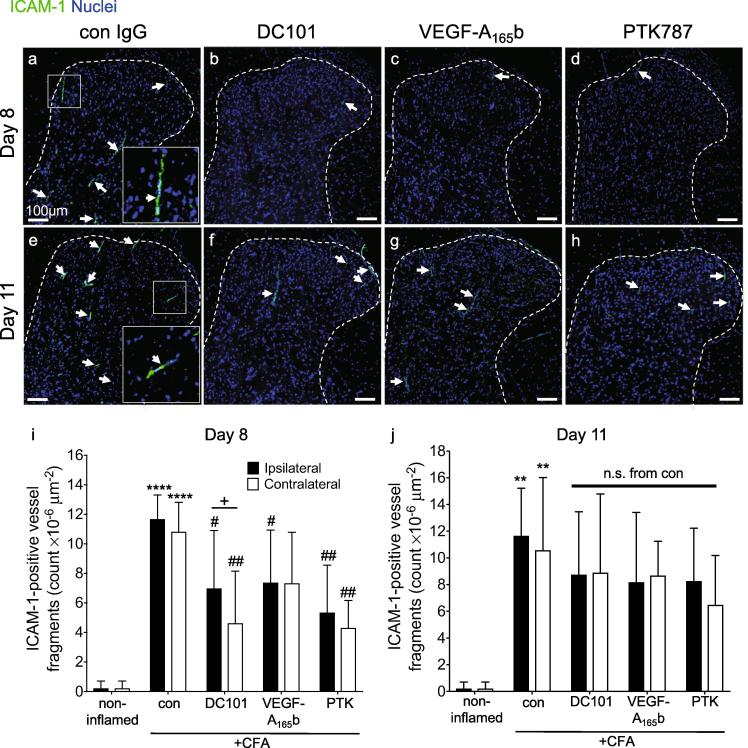
The effect of anti-VEGFR2 agents on dorsal horn vascular activation. Representative low magnification images of ICAM-1 immunoreactivity in the dorsal horn (a-h). Number of ICAM-1^+^ microvessels present in the dorsal horn (i,j). Three statistical analyses were performed: 2-way analysis of variance + Dunnett’s multiple comparisons test: control vs. non-inflamed *p < 0.5, **p < 0.01, ***p < 0.001, ****p < 0.0001; anti-VEGFR2 agent vs. control (ipsi or contra): #*p* < 0.5, ##*p* < 0.01, ###*p* < 0.001, ####*p* < 0.0001 and ipsi vs. contra of respective group +p < 0.5. Day eight: n = 4; day 11: n = 4–5. Data displayed as mean ± SD.

Inhibiting VEGF-A can inhibit ICAM-1 expression and monocyte adhesion *in vitro* (Radisavljevic et al., 2000, Thichanpiang et al., 2014). CD11b^+^ cells were initially associated with microvessels (day eight) and were later located in the dorsal horn parenchyma (day 11). We therefore tested the hypothesis that peripheral inflammation caused an increase in dorsal horn endothelial ICAM-1 expression which could underpin the possible transendothelial migration of CD11b^+^ cells into the parenchyma. Compared with control animals, ICAM-1^+^ vessels in CFA-treated animals were present throughout the dorsal horn after 8 days Fig. 5a), whereas fewer were seen in DC101, VEGF-A_165_b or PTK787-treated groups at day eight Fig. 5b-d) but not at day 11 Fig. 5e-h). Quantification of the number of ICAM^+^ vessels showed that CFA induced a significant increase on both sides of the spinal cord at day eight, which was significantly reduced by DC101, VEGF-A_165_b or PTK787 Fig. 5i). While the ICAM-1 induction was still evident at day 11 Fig. 5j), VEGFR inhibition had no effect at this time point. There were no differences between the ipsi- and contralateral sides within each group except in the DC101 group (day 8, Fig. 5i). The significant inhibition of ICAM-1^+^ microvessels by anti-VEGFR2 treatment at day 8 may have reduced CD11b^+^ microglia-like cell transmigration into the parenchyma Fig. 4 m).

### 3.5 The effect of VEGFR2 inhibitory agents on dorsal root ganglion vascular ICAM-1 expression

**Fig. 6:**
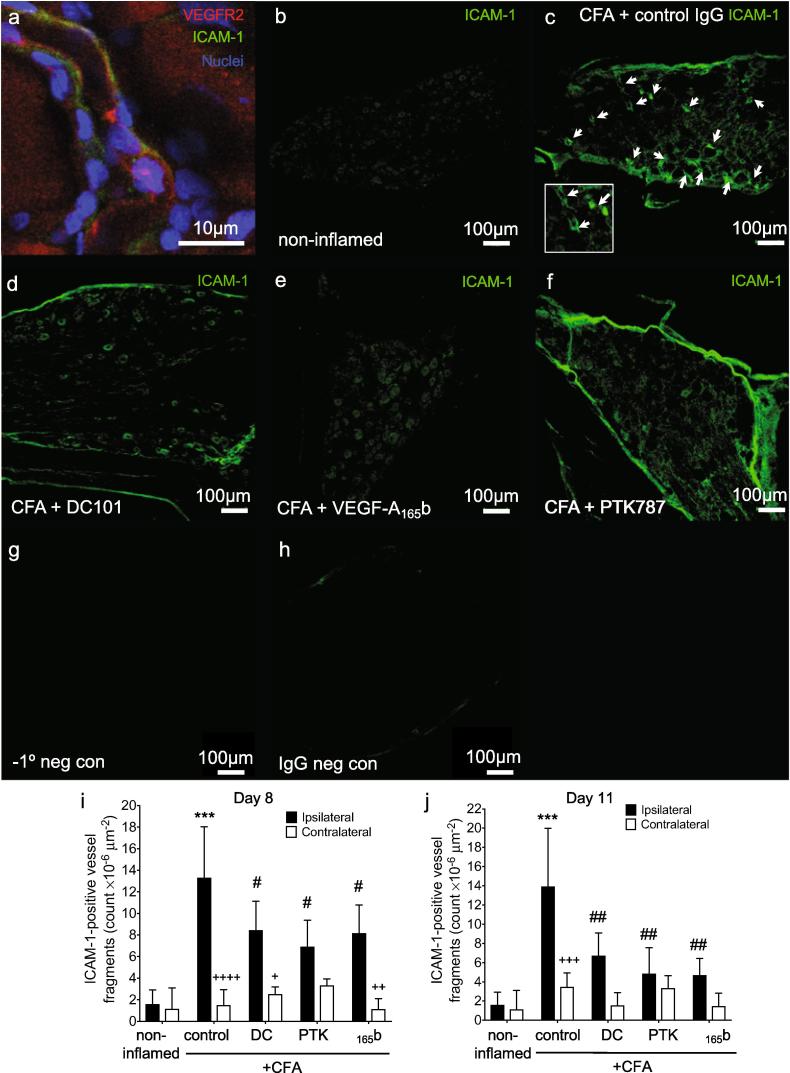
VEGFR2 inhibition reduces ICAM-1 expression in DRG. ICAM-1 and VEGFR2 immunoreactivity in the ipsilateral L3 dorsal root ganglion eight days following induction of inflammatory arthritis (a). ICAM-1 expression was localized to DRG microvessels expressing VEGFR2 (scale bar- 10 μm). Representative images showing ICAM-1 expression in L3 DRG 8 days following induction of inflammatory arthritis with treatment with control IgG, DC101, VEGF-A_165_b or PTK787 (b-f). Arrow indicates ICAM-1^+^ vessel fragment. No primary and species- and concentration-matched isotype control IgG staining (g,h). Scale bar-100 μm. Quantification of microvessel ICAM-1 expression in ipsilateral and contralateral L3 DRG from non-inflamed, or CFA injected animals on day eight (i) and 11 (j) and with anti-VEGF treatments. Statistical analyses: g,h: one-way analysis of variance + Dunnett’s multiple comparisons test: vs. non-inflamed *p < 0.5, **p < 0.01, ***p < 0.001, ****p < 0.0001; vs. CFA + IgG control: #p < 0.5, ##p < 0.01, ###p < 0.001, ####p < 0.0001 and ipsi vs. contra of respective group +p < 0.05, ++p < 0.01, +++p < 0.001, ++++p < 0.0001. Day eight: n = 3–4; day 11: n = 4–5. Data displayed as mean ± SD.

Infiltration of inflammatory cells into DRG contributes to chemotherapy-induced pain (Zhang et al., 2016), diabetic neuropathy (Rahman et al., 2016), and traumatic nerve injury (Austin et al., 2015). ICAM-1/VEGFR2 double staining demonstrated that both molecules were expressed in DRG of animals with CFA monoarthritis Fig. 6a). ICAM-1 staining was noticeably more frequent in ipsilateral DRG from CFA-induced animals Fig. 6b,c) and this was less so with DC101 Fig. 6d), VEGF-A_165_b Fig. 6e) and PTK708 Fig. 6f) treatment. There was a significant increase in the number of ICAM-1^+^ vessels in ipsilateral, but not contralateral L3 DRG compared to non-inflamed control after eight Fig. 6i) and 11 days Fig. 6j). All three VEGFR2 inhibitory treatments significantly decreased the number of ICAM-1^+^ vessels at both eight and 11 days Fig. 6 g,h). No macrophages (CD68^+^ cells) were observed in the DRG on day eight in any group (data not shown).

### 3.6 Targeting VEGFR2 in cultured human brain microvascular endothelial cells in a fluorescent monocyte adhesion assay

**Fig. 7:**
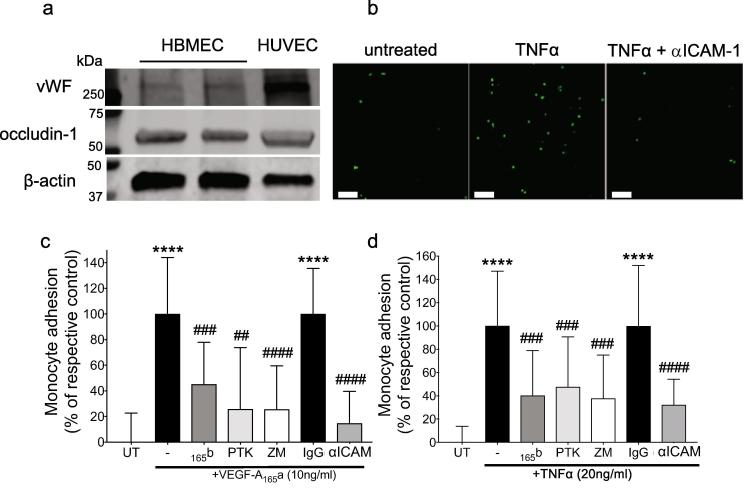
Targeting VEGFR2 reduced monocyte adhesion to brain endothelial cells *in vitro*. Expression of endothelial associated protein von Willebrand Factor, and occludin-1, in two different primary human brain microvascular endothelial cells (HBMEC) cultures and HUVEC (a). Representative images of fluorescent monocytes attached to cultured brain endothelial monolayer following treatment with or without TNF-α or TNF-α+ ICAM-1 blocking antibody, scale bar = 100 μm (b). Effect of pre-treatment with a selective VEGFR2 inhibitor ZM883231 (ZM, 20 nM, 1 h), VEGF-A_165_b (10 ng/ml, 24 h), the VEGFR1&2 inhibitor PTK787 (PTK, 100 nM, 1 h) or an ICAM-1 blocking antibody (conc, time) or non-specifc IgG on VEGF-A_165_a-induced monocyte adhesion, n = 9 (c). Effect of pre-treatment with VEGF-A_165_b (24 h), PTK787 (1h), an ICAM-1 blocking antibody or ZM323881 (1h) on TNF-α-induced monocyte adhesion (d). Statistical analyses: 1-way analysis of variance + Dunnett’s multiple comparisons test: vs. untreated *p < 0.5, **p < 0.01, ***p < 0.001, ****p < 0.0001; vs. respective control: #p < 0.5, ##p < 0.01, ###p < 0.001, ####p < 0.0001. Abbrev. Ab – antibody; HBMEC – human brain microvascular endothelial cells; HUVEC – human umbilical vein endothelial cells; vWF – von Willebrand Factor; VEGF-A – vascular endothelial growth factor-A; TNF-α – tumor necrosis factor-α; ICAM-1 – intercellular adhesion molecule-1. Adhesion assays: n = 9–20 (individual wells collated from three independent experiments). Data displayed as mean ± SD.

VEGF-A_165_a and TNF-α upregulate ICAM-1 expression through activation of VEGFR2 in endothelial cells (Radisavljevic et al., 2000, Hirano et al., 2017), retinal pigmented epithelial cells (Thichanpiang et al., 2014, Lee et al., 2015), and rheumatoid arthritis synoviocytes (Hulse et al., 2015, Lindsley et al., 1993). To test the hypothesis that effects on ICAM-1 upregulation and monocyte attachment were attributable to a possible direct action of VEGFR2 inhibitory agents on CNS endothelium we used human brain microvascular endothelial cell (HBMEC) monolayers. An endothelial phenotype was confirmed in HBMEC by immunoblot for von Willebrand factor (vWF, Fig. 7a, HUVEC-positive control). These cells also expressed occludin, indicating the formation of tight junctions. Cells treated with increasing concentrations of TNF-α showed that fluorescently labeled monocyte (THP-1) attachment to HBMEC monolayers was significantly induced by TNF-α treatment and this was significantly reduced by co-treatment with an ICAM-1 blocking antibody Fig. 7b). Treatment with VEGF-A_165_a (24 h) also caused a significant increase in fluorescence THP-1 monocyte attachment Fig. 7c), and VEGF-A_165_b pre-treatment significantly inhibited this attachment Fig. 7c). Pre- and co-treatment with a specific VEGFR2 inhibitor (20 nM ZM323881, Fig. 7d,e) or PTK787 (100 nM, Fig. 7d) also significantly inhibited VEGF-A_165_a-induced THP-1 monocyte attachment to HBMEC, as did the addition of an ICAM-1 blocking antibody Fig. 7c). In a similar manner to VEGF-A_165_a, TNF-α treatment (24 h) caused an increase in attached THP-1 monocyte fluorescence Fig. 7d) that could be blocked with VEGF-A_165_b, anti-VEGFR2 compounds or blocking ICAM-1 antibody Fig. 7d). Thus TNF-α-mediated THP-1 monocyte adherence to endothelial cells from central nervous tissue appears to be VEGFR2-dependent.

### 3.7 Characterisation of Tie2CreER^T2^-mediated VEGFR2 knock-out

Before commencing a peripheral inflammatory pain model we characterized the VEGFR2 knock-out in lung and spinal cord. The VEGFR2 genomic KO product was detected only in CD31^+^ cells from tamoxifen-treated Tie2CreER^T2^ *vegfr2*^fl/fl^ mice, and not (vehicle-treated Tie2CreER^T2^ *vegfr2*^fl/fl^ (uninduced) mice or Tie2CreER^T2^-negative (wildtype) mice, indicating VEGFR2 endothelial cell knock-out (VEGFR2^ECKO^) (Supplementary Fig. 2). Tie2 and VEGFR2 expression levels were analysed by flow cytometry in CD31^+^ lung (Supplementary Fig. 2) and CD31^+^ spinal cord cells (Supplementary Fig. 3). In both samples VEGFR2^ECKO^ significantly reduced the number of viable Tie2^+^ cells (lung, reduced by 21.1 ± 6.3%, spinal cord reduced by 42.6 ± 8.3%) and the number of Tie2^+^ cells expressing VEGFR2 (lung reduced by 48 ± 6.9%, spinal cord 45.4 ± 12.1%) (Supplementary Figs. 2&3).

### 3.8 Inducible Tie2CreER^T2^-mediated VEGFR2 knock-out affected the development of pain-associated behaviors in a model of inflammatory arthritis

**Fig. 8:**
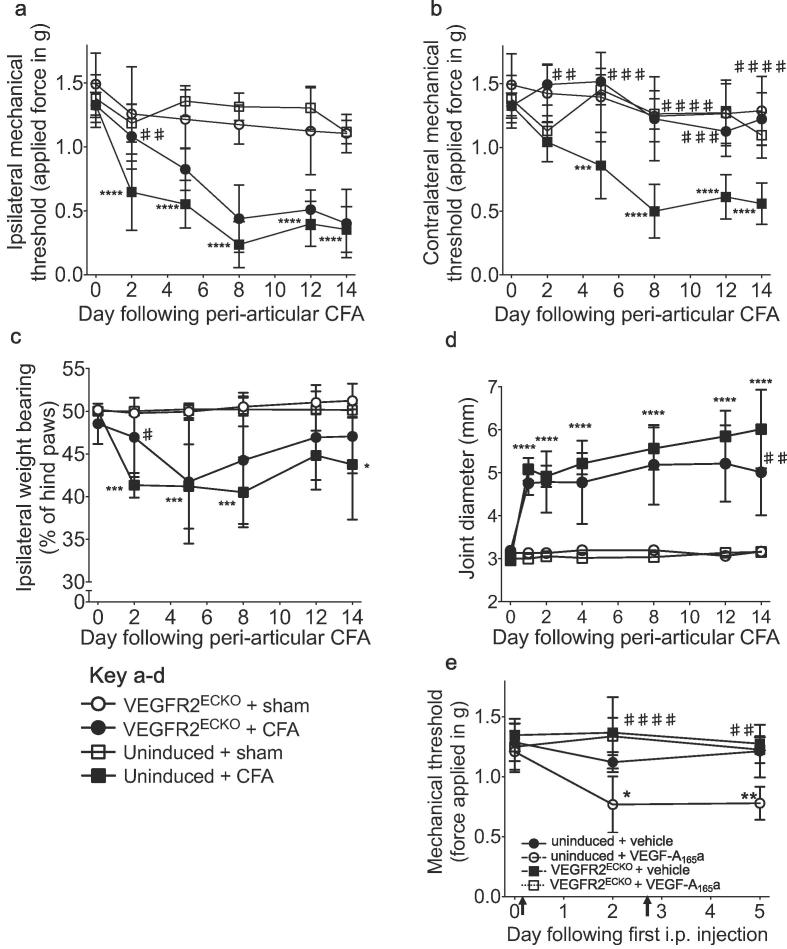
Endothelial VEGFR2 knock-out affected pain behaviors in comparison to uninduced control animals. Ipsilateral (a) and contralateral (b) hind paw mechanical stimulus threshold in control (uninduced) animals. In VEGFR2^ECKO^ mice, the reduction in mechanical stimulus threshold was significantly delayed in (a) the ipsilateral hind paw and significantly prevented in (b) the contralateral hind paw in comparison with uninduced control mice. A significant inhibition of the CFA-induced shift in weight bearing was also observed on day 2 (c) and by day 14 there was a significant reduction in joint diameter (d). Mechanical allodynia 2 and 5 days following the start of recombinant human VEGF-A_165_a treatment (8ng/g i.p. biweekly) (g). Statistical analyses: b-f, h: 2-way repeated measures analysis of variances + Dunnett’s multiple comparisons test; vs. respective baseline (day 0): *p < 0.5, **p < 0.01, ***p < 0.001, ****p < 0.0001; vs. respective control at given time point: ##*p* < 0.01, ###*p* < 0.001, ####*p* < 0.0001; f: Student’s *t*-test, *p < 0.05. a: n = 3; b: n = 10–11; c-f n = 5–6; g,h; n = 5. Abbrev. CFA, complete Freund’s adjuvant. Data displayed as mean ± SD.

**Fig. 9:**
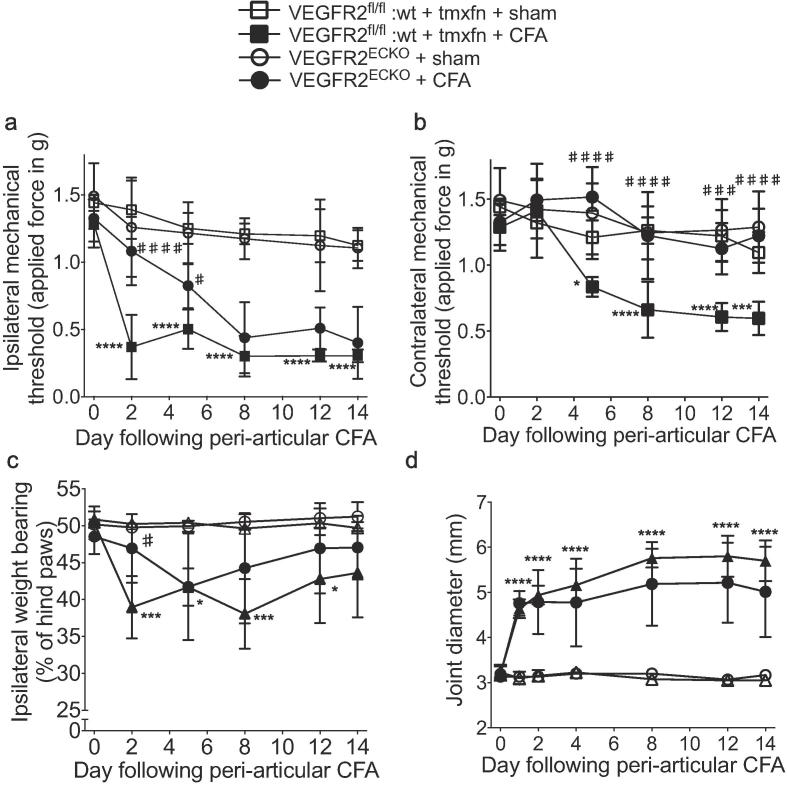
Endothelial VEGFR2 knock-out affected pain behaviors in comparison to tamoxifen-treated Tie2CreER^T2^-wildtype control animals. In addition to the knock-out and uninduced experimental groups (refer to Fig. 8, a tamoxifen-treated Tie2CreER^T2^-negative (wildtype) group was included in the *peri*-articular CFA experiment. A significant reduction in ipsilateral (a) and contralateral (b) mechanical stimulus threshold developed in these mice as was observed in the uninduced control group (refer to Fig. 8. These effects were significantly different from the knockout group (ipsi: day 2, contra: all time points). A significant inhibition of the CFA-induced shift in weight bearing was also observed on day 2 (c) while no difference was observed in joint swelling (d). Statistical analyses: two-way repeated measures analysis of variances + Dunnett’s multiple comparisons test; either control vs. respective baseline (day 0): *p < 0.5, **p < 0.01, ***p < 0.001, ****p < 0.0001; or test groups vs. respective control at given time point: ##*p* < 0.01, ###*p* < 0.001, ####*p* < 0.0001, n = 5–6. Data displayed as mean ± SD.

Hind paw mechanical stimulus thresholds were not affected by tamoxifen treatment in either VEGFR2^ECKO^ or *vegfr2*^fl/fl^:wt mice compared with baseline or respective vehicle-treated control groups over a 2-week period following the start of dosing (Supplementary Fig. 4a). CFA inflammation was induced in VEGFR2^ECKO^ and the two control groups [*vegfr2*^fl/fl^:Tie2CreER^T2^ mice treated with vehicle (uninduced, Fig. 8) and *vegfr2*^fl/fl^:wt treated with tamoxifen, Fig. 9] by *peri*-articular ankle joint injection. CFA caused a significant drop in the ipsilateral hind paw mechanical stimulus threshold measured in both control mouse groups from day two onwards compared with respective baseline or sham injection Figs. 8a & 9a). In VEGFR2^ECKO^ mice, CFA caused a significant reduction in ipsilateral mechanical stimulus threshold only from day five onwards which was significantly different from the uninduced group on day two Fig. 8a) and the *vegfr2*^fl/fl^:wt + tamoxifen group on days two and five Fig. 9a). CFA also caused a significant reduction in the contralateral hind paw mechanical threshold measured in control mouse groups from day five onwards compared to respective baseline or sham control. In contrast, VEGFR2^ECKO^ mice did not develop contralateral mechanical sensitivity Figs. 8b & 9b). CFA caused a significant shift in weight bearing to the contralateral hind paw in uninduced control mice compared to respective sham animals on days two, five, eight and 14 yet a significant shift was observed in the VEGFR2^ECKO^ animals only on day five. On day two there was a significant difference between VEGFR2^ECKO^ and both control groups Figs. 8c & 9c). All three groups developed a significant increase in the ipsilateral tibiotarsal joint diameter following CFA over the course of the experiment Figs. 8d &. 9d) and a significant difference was observed only on day 14 between VEGFR2^ECKO^ and uninduced mice Fig. 8d). Following the completion of the behavioral assessment, the level of VEGFR2 mRNA was determined in CD31^+^ lung cells from the same mice to confirm continued knock-out. VEGFR2 mRNA detected in KO mice was significantly reduced (by 57.2 ± 4.3% of uninduced mice, n = 5, Supplementary Fig. 4b).

In a separate experiment, knock-out and control mice were treated with rhVEGF-A_165_a [8ng/g body weight i.p., known to result in hind paw mechanical allodynia (Hulse et al., 2014)] 2 weeks after tamoxifen treatment. Biweekly rhVEGF-A_165_a treatment caused a significant reduction in hind paw mechanical threshold Fig. 8e). In contrast, biweekly treatment of rhVEGF-A_165_a in knock-out mice had no effect on mechanical threshold and this was significantly different from control mice at the two time points tested Fig. 8e).

**Fig. 10:**
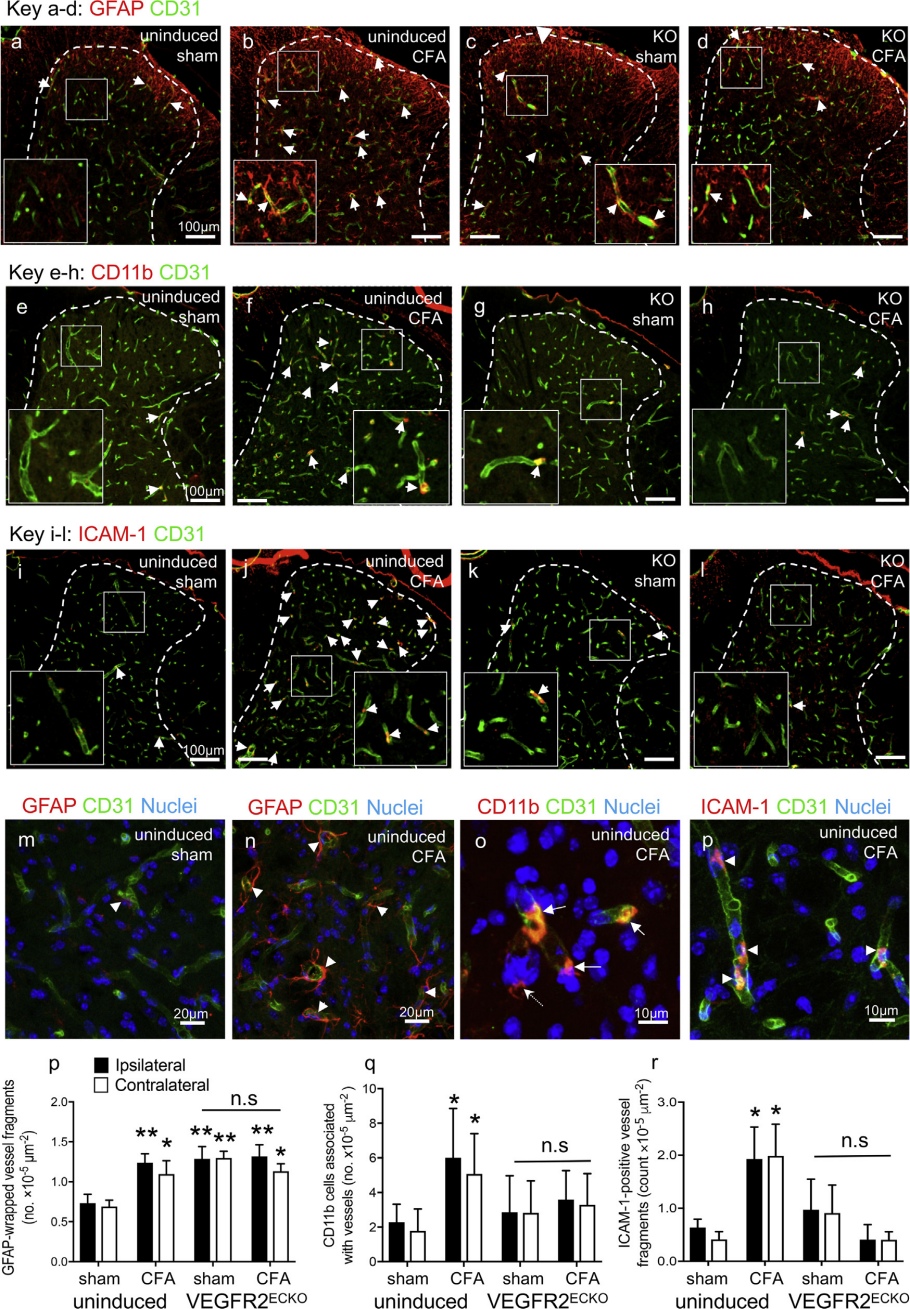
VEGFR2^ECKO^ inhibited glio-vascular activation in the dorsal horn of CFA treated mice. Peri-articular CFA caused an significant increase in the number of dorsal horn CD31^+^ vessels associated with GFAP^+^ reactive astrocytic foot processes (glio-vascular response) compared with sham in uninduced mice (a,b, quantification in p). There was a significant increase in the number of vessels associated with GFAP^+^ astrocytic foot processes in sham-treated VEGFR2^ECKO^ compared with sham-treated uninduced controls, however CFA treatment in VEGFR2^ECKO^ mice did not significantly increase this further (c,d, quantification in p). CFA caused a significant increase in the number of CD11b^+^ cells associated with CD31^+^ vessels and the number of ICAM-1^+^ vessel structures in uninduced mice compared with sham (e,f,i,j quantification in q&r) but the same effects were not observed in VEGFR2^ECKO^ (g,h,k,l quantification in q&r). Higher magnification images of GFAP^+^ reactive astrocytic end feet (m, n, arrowheads denote GFAP^+^/CD31^+^ vessel structures), CD11b^+^ cells associated with vessel (n, arrows denote CD11b^+^ cells associated with CD31^+^ vessel structures, dotted arrow denotes a parenchyma CD11b^+^ cell) and ICAM-1^+^ vessels (o, arrowheads denote ICAM-1^+^/CD31^+^ vessel structures). Three statistical analyses were performed: 2-way ANOVA + Bonferroni multiple comparisons test: vs. uninduced sham con *p < 0.05, ** *p* < 0.01; KO CFA vs KO sham control – no significance; contra vs. ipsi of respective group – no significane; n = 3–6. Data displayed as mean ± SD.

Following the completion of the VEGFR2^ECKO^ inflammatory arthritis behavioral assay we investigated expression levels of GFAP, ICAM-1 and CD11b in the dorsal horn (day 14 tissue). Peri-articular CFA caused an significant increase in the number of dorsal horn CD31^+^ vessels associated with GFAP^+^ reactive astrocytic end-feet (glio-vascular response) compared with sham uninduced mice Fig. 10a,b,m,n, quantification in p). There was also a significant increase in the number of vessels associated with GFAP^+^ astrocytic end-feet in VEGFR2^ECKO^ mice compared with sham uninduced controls, however CFA treatment in VEGFR2^ECKO^ mice did not significantly increase this further Fig. 10c,d, quantification in p). CFA caused a significant increase in the number of CD11b^+^ cells associated with CD31^+^ vessels and the number of ICAM-1^+^ / CD31^+^ vessels in uninduced mice compared with sham Fig. 10e,f,i,j,o-r) but the same effects of CFA were not observed in VEGFR2^ECKO^ mice Fig. 10 g,h,k,l quantification in q&r). There were no significant differences between the ipsi- and contralateral sides within each group in either of the analyses. A low number of CD11b^+^ cells were detected in the dorsal horn parenchyma and CFA did not significantly affect this (Supplementary Fig. 5).

## 4 Discussion

Pharmacological targeting of VEGFR2 affects nociception in a number of preclinical models of pain (Hulse et al., 2014, Hulse et al., 2015, Hulse et al., 2016, Nesic et al., 2010, Lin et al., 2010, Nagai et al., 2014), but the cellular location of the VEGFR2 involved in this process and the underlying mechanism have not been clearly elucidated. Here we provide evidence that inhibiting *endothelial* VEGFR2 disrupts the development of secondary pain behavior in rats (systemic pharmacological inhibition including an anti-VEGFR2 antibody that would not freely cross the BBB/BSCB, Fig. 2) and in inducible endothelial VEGFR2 knock-out mice (Fig. 9). Conversely, targeting *local* VEGFR2 by intra-articular injection of anti-VEGFR2 agents (Fig. 3) did not affect secondary mechanical allodynia indicating that the anti-nociceptive effects of *systemic* VEGFR2 inhibition are not elicited by an inhibitory mechanism at the site of inflammation in the joint. The inhibition of secondary pain-like behaviors (the spread of sensitivity) indicates an endothelial site of action in the PNS and/or CNS. Systemic anti-VEGFR2 treatment reduced endothelial activation and the number of CD11b^+^ microglia-like cells in the lumbar spinal cord dorsal horn parenchyma (Figs. 4 and 5) and endothelial activation in the DRG (Fig. 6) in response to knee joint inflammation. Furthermore inducible endothelial knock-out prevented glio-vascular activation in response to ankle joint inflammation. These results highlight that spinal and DRG microvasculature may contribute to the development of nociceptive processes in peripheral inflammation, and indicate the importance of endothelial VEGFR2 in the spread of pain. Further work using this model could investigate whether the observed spinal cord glio-vascular response is restricted to segments corresponding to the inflamed joint or is more widespread within the CNS. The outcome would indicate either a sensory afferent-mediated neurogenic glio-vascular response or CNS glio-vascular activation in response to systemic inflammation. The absence of ICAM-1^+^ upregulation in the contralateral DRG indicates that the observed spinal glio-vascular activation in both sides of the cord was in response to CFA-induced ipsilateral peripheral drive as opposed to a widespread vascular response to systemic inflammation. The significant effects observed in the contralateral spinal cord are likely to be a consequence of commissural neuronal signaling which includes somatosensory/nociceptor signaling (Comer et al., 2015).

Reactive glia and monocyte/macrophage infiltration in the CNS are common features of chronic neurodegenerative (Rezai-Zadeh et al., 2009) and autoimmune diseases (Winer, 2001, Ziff, 1989), and CNS injuries such as stroke (Gronberg et al., 2013, Michell-Robinson et al., 2015). Reactive astrocytes and microglia-like cells are found in the spinal dorsal horn in patients with longstanding complex regional pain syndrome (Del Valle et al., 2009). In rat peripheral sciatic nerve injury, a significant proportion of dorsal horn cells expressing the migroglial marker Iba1 were found to derive from circulating monocytes/macrophages that had migrated into the spinal cord parenchyma and differentiated into a reactive microglia-like phenotype (Zhang et al., 2007, Echeverry et al., 2011). For this process to occur, the spinal cord endothelium needs to be stimulated to express adhesion molecules for monocyte transmigration across the BSCB. Furthermore both residential microglia and peripheral monocytes are required for the transition from acute to chronic pain after nerve injury providing further evidence that peripheral immune cells are involved in the development of chronic pain (Peng et al., 2016). Our findings suggest transendothelial migration of CD11b^+^ monocytes into the spinal dorsal horn parenchyma occurs following peripheral inflammation and is controlled by vascular VEGFR2-mediated ICAM-1-induced adhesion. Circulating monocytes also transmigrate across the blood-nerve barrier in sensory ganglia and take on a macrophage-like phenotype, in a preclinical inflammatory pain model (Segond von Banchet et al., 2009), and CD68^+^ macrophages migrate into DRG in antigen-induced arthritis progression (Jochmann et al., 2015). We did not observe CD68^+^ cells in DRG despite increased vascular ICAM-1 expression. Our findings on GFAP, CD11b and ICAM-1 expression in both sides of the spinal cord and ipsilateral DRG indicate that nervous system-endothelial VEGFR2 promotes endothelial ICAM-1 expression in the dorsal horn and DRG in inflammatory arthritis. CD11b is expressed on reactive microglia (Sedgwick et al., 1998) and also neutrophils, monocytes, natural killer cells and subsets of lymphocytes (Springer, 1990, Kawai et al., 2005). CD11b contributes to firm leukocyte adhesion to activated endothelium via interaction with ICAM-1, sub-endothelium basement membrane components and pericytes (Thompson and Matsushima, 1992, Lundgren-Akerlund et al., 1993, Bohnsack et al., 1990, Ding et al., 1999, Schmitt et al., 2018). The reduction of CD11b^+^ cells in the rat spinal cord parenchyma by day 11 suggests that the reactive microvasculature contributes to transmigration of CD11b^+^ cells into the parenchyma and/or the activation of residential microglia through a VEGFR2-mediated mechanism. In stroke models, brain pericytes release from the microvasculature, migrate into the parenchyma and differentiate into a CD11b^+^ microglia-like phenotype (Ozen et al., 2014). In the present study dorsal horn glio-vascular activation could trigger a similar process providing an alternative source for the increase in CD11b^+^ cells within the dorsal horn parenchyma. In our inducible endothelial VEGFR2 KO study a significant increase in the number of parenchymal microglia-like cells was not detected in the dorsal horn 14 days after CFA administration in uninduced control mice. However a significant increase in the number of CD11b^+^ cells associated with vessels and ICAM-1^+^ vessel structures were both observed, which endothelial VEGFR2 KO prevented. These results implicate vessel-associated CD11b^+^ cells and ICAM-1 vessel expression in the central changes that occur following peripheral inflammation and provide further evidence that endothelial VEGFR2 may drive a vascular mechanism that promotes the development of secondary pain. In our rat study the number of CD11b^+^ cells associated with the dorsal horn microvasculature on day 8 was not different between the inflamed groups suggesting that the anti-VEGFR2 treatments did not affect the maturation and release of immune cells into the circulation through an inhibitory mechanism in bone marrow. Furthermore we could not detect a significant population of Tie2^+^/VEGFR2^+^ peripheral mononuclear cells in the uninduced mice (data not shown) and VEGFR2^ECKO^ knock-out mice also show no change in the number of CD11b^+^ cells isolated from spleen (Ved et al., under review). Therefore we think the KO is unlikely to have caused a decrease in circulating CD11b^+^ immune cells that consequently reduced CD11b^+^ cell transmigration into the spinal parenchyma, thus supporting an endothelial site of action of anti-VEGFR2. The *in vitro* inhibition of THP-1 monocyte adhesion to brain microvascular endothelial cells by anti-VEGFR2 compounds supports the hypothesis that *in vivo* VEGFR2 inhibition may reduce CD11b^+^ monocytic adhesion and infiltration or pericytic migration into the spinal cord through a reduction in VEGF-A-mediated vascular activation and ICAM-1 expression.

VEGF-A protein is increased in the serum of rheumatoid arthritis patients (Nakahara et al., 2003), and is upregulated in painful disease models such as chronic constriction injury (Lin et al., 2010, Tang et al., 2009) and type I diabetes (Samii et al., 1999). Inhibition of VEGFR2 signaling decreases pain-associated responses (Lin et al., 2010). VEGF-A_165_b, a splice isoform of VEGF-A is a partial agonist of vascular endothelial growth factor receptor-2 (VEGFR2) and competes with VEGF-A_165_a for binding to VEGFR2 (Kawamura et al., 2008). Systemic VEGF-A_165_b administration is also able to reduce neuronal damage and pain-associated responses in nerve injury (Hulse et al., 2014, Beazley-Long et al., 2013) and type I diabetes models (Hulse et al., 2015). Intrathecal blockade of VEGF-A or VEGFR2 inhibits pain responses in both pain models and normal animals (Zhang et al., 2011, Hess et al., 2011, Boettger et al., 2010), and intrathecal delivery of rhVEGF-A_165_a is pro-nociceptive in normal animals (Hulse et al., 2016) indicating central nociceptive actions of VEGF-A/VEGFR2. Combined with our findings, there is compelling evidence that systemic anti-VEGFR2 therapy could be of analgesic benefit to patients with inflammatory arthritis, even when disease is controlled by anti-TNF-α treatment.

The time points at which we observed activated vessels are consistent with the spinal endothelial activation being a consequence of nociceptor-driven central changes, as opposed to an acute systemic inflammatory response, as both increased peripheral drive (Schaible and Schmidt, 1988, Andrew and Greenspan, 1999) and central sensitisation (Neugebauer and Schaible, 1988) occur within hours. Furthermore the increase in the number of GFAP^+^ astrocytic end-feet wrapping ICAM-1^+^ vessels in the dorsal horn and the timing of these changes provides a likely astrocyte-mediated mechanism for microvessel activation in response to inflammation-driven neuronal input. Such glia-vascular co-activation, with ICAM-1 expression, is also found in specific brain regions in acute inflammation, along with immune cell infiltration and is suggested to contribute to breakdown of blood-brain barrier integrity in inflammatory pain (Willis et al., 2008, Huber et al., 2006). Our findings are the first to show a function for endothelial VEGFR2 in peripheral inflammation-driven glio-vascular activation. If depleting circulating monocytes produces a similar anti-nociceptive effect to anti-VEGFR2 in inflammatory arthritis this would provide direct evidence of transmigrating pro-nociceptive immune cells that is mediated by VEGFR2.

The acute anti-nociceptive effect of anti-VEGFR2 treatment (<8 days) cannot be attributable to inhibition of transendothelial migration in the dorsal horn as this preceded the increase in CD11b^+^ microglia in the dorsal horn. Systemic and local injection of rhVEGF_165_a can directly drive nociceptor activity (Hulse et al., 2014). It is possible therefore that the acute anti-nociceptive effect of systemic anti-VEGFR2 was a consequence of direct sensory neuronal inhibition (Hulse et al., 2014), and/or an indirect anti-nociceptive effect through the inhibition of endothelial VEGFR2 in sensory nerve, ganglia and/or CNS at a time prior to day eight. In the present study inducing endothelial VEGFR2 knock-out prevented the acute pro-nociceptive effect of systemic rhVEGF-A_165_a in the absence of inflammation. When considered with the anti-nociceptive effect of receptor inhibition, these findings lead us to conclude that VEGF-A_165_a working through endothelial VEGFR2 and endothelial activation, is involved in the initial nociceptive processes underpinning the development of secondary hypersensitivity, as well as spinal vascular activation and possible transendothelial migration of immune cells into the dorsal horn.

VEGFR2 knock-out is embryonically lethal due to severe vascular defects (Shalaby et al., 1995) therefore to eludicate the site of action of anti-VEGFR2 we generated and characterized an inducible, Tie2-specific VEGFR2 knock-out mouse, enabling the targeting of endothelial VEGFR2 in the adult mouse in a peripheral inflammation pain study. First we characterised the inducible Tie2CreER^T2^ VEGFR2 knock-out at DNA, RNA and protein levels in the lung, and protein level in the spinal cord revealing a significant reduction of VEGFR2 expression in endothelial (CD31^+^/Tie2^+^) cells. VEGFR2^ECKO^ caused a significant reduction in viable CD31^+^/Tie2^+^ cells (in both lung and spine) indicating an effect on endothelial viability. VEGFR2^ECKO^ also caused a significant reduction in VEGFR2 expression by CD31^+^/Tie2^+^ cells (both lung and spine). The reduction in endothelial VEGFR2 expression induced by the knock-out is therefore likely to be significantly underestimated, as a result of additional loss of Tie2^+^/VEGFR2^+^ cells. The VEGFR2^ECKO^ knock-out mice also display aberrant spinal cord microvasculature morphology with a decrease in microvessel diameter and volume (Ved et al., under review) supporting a survival role for VEGFR2 in the adult transgenic mice.

## 5 Conclusions

Inflammatory arthritis causes glio-vascular activation, endothelial ICAM-1 expression and an increase in parenchymal CD11b^+^ microglia-like cells in the dorsal horn of the lumbar spinal cord. Furthermore targeting endothelial VEGFR2 is anti-nociceptive in inflammatory arthritis, and reduces vascular activation and the number of CD11b^+^ microglia-like cells in the dorsal horn. The association between the reduction of dorsal horn endothelial activation and parenchymal CD11b^+^ cells, and the spread of mechanical sensitivity has led us to propose a novel nociceptive mechanism: spinal cord glio-vascular activation promotes transendothelial migration of pro-nociceptive immune cells into neural parenchyma and contributes to central sensitization and the spread of inflammatory pain. In addition we hypothesise that increased VEGF-A levels in the serum of people with rheumatoid arthritis could activate neural microvasculature and promote transmigration of immune cells, thus contributing to central sensitization and potentially the development of a chronic pain state. Our results indicate that VEGFR2 inhibitors to prevent vascular action and immune cell translocation could be a new additional therapeutic strategy for rheumatoid arthritis patients as an adjunct to existing disease-modifying therapies.

## Appendix A Supplementary data

Supplementary data 1 Correlation analyses of CD11b^+^ dorsal horn parenchymal cell number and ICAM-1^+^ vessel number versus ipsilateral mechanical stimulus withdrawal threshold. The significant increase in dorsal horn CD11b^+^ cells induced by intra-articular CFA significantly and negatively correlated with the previous day’s reduced hind paw mechanical threshold (a). There was also a significant and negative association between dosal horn (b,c) and DRG (d,e) ICAM-1^+^ vessels and ipsilateral mechanical threshold at both time points. Statistical analyses: Spearman’s correlation test, n = 18–21.

Supplementary data 2 Tamoxifen-induced Tie2CreER^T2^ VEGFR2 knock-out was investigated in primary CD31^+^ lung cells. The VEGFR2 knock-out gene product was detected only in CD31^+^ lung cells and not the flow-through fraction from tamoxifen-dosed VEGFR2^fl/fl^:Tie2CreER^T2^-positive mice indicating the VEGFR2 knock-out is inducible by tamoxifen and specific for CD31^+^ cells (n = 3) (a). Two distinct populations of living CD31^+^ cells (b; calcein^+^) were identified by scatter profile (c). A low Tie2/VEGFR2-negative (non-endothelial) population (d), and a high Tie2/VEGFR2-expressing (endothelial) population (e). An example of the endothelial population Tie2/VEGFR2 in KO mice (f). The number of Tie2^+^ cells in the endothelial population in VEGFR2^ECKO^ and littermate control (d). Control stains using either Tie2 or VEGFR2 antibody alone revealed no channel compensation was required (g,h). Number of Tie2^+^ cells in the non-endothelial population (i). Percentage of Tie2^+^ cells that were VEGFR2^+^ in the endothelial population (j). Percentage of total endothelial population that were Tie2^+^/VEGFR2^+^ (k). VEGFR2 median fluorescence value of all Tie2^+^ cells within the endothelial population (l). Statistical analyses: student’s t-test: **p* < 0.5, ***p* < 0.01. Data presented as mean± SD, n = 5–6.

Supplementary data 3 Investigating the level of endothelial VEGFR2 knock-out by flow cytometry in the CD31^+^ spinal cord cells. No distinct populations of living spinal cord CD31^+^ cells (a; calcein^+^, Hoechst^+^) were identified by scatter profile (data not shown) so all living cells CD31^+^ were analysed. An artefactual population, possibly contaminating myelin, displayed properties not consistent with cells (a). An example of the Tie2/VEGFR2 in uninduced mice (b) and VEGFR2^ECKO^ mice (c). Control stains using either Tie2 or VEGFR2 antibody alone revealed no channel compensation was required (d,e). Viable CD31^+^ Tie2^+^ cells as fold change of wildtype control (f) and VEGFR2^+^/Tie2^+^ of Tie2^+^ population as a fold change of wildtype control (g). Statistical analyses: 1-way ANOVA + Dunnett’s multiple comparisons test: vs. wildtype control, **p* < 0.5, ***p* < 0.01, n = 5–8. Data presented as mean ± SD.

Supplementary data 4 VEGFR2^ECKO^ did not affect mechanical threshold in uninflamed mice and caused a long lasting reduction in VEGFR2 mRNA in CD31^+^ lung cells. Treatment with tamoxifen or its vehicle had no effect on mechanical stimulus threshold in either VEGFR2^ECKO^, uninduced or wild type (wt) mice up to 2 weeks following the start of tamoxifen dosing (a). Following the completion of the ankle joint behavioral assessment (4 weeks after tamoxifen treatment) the level of VEGFR2 mRNA in CD31^+^ cells from knock-out mice was 57% lower compared with uninduced control indicating a long-lasting effect of the knock-out. Measured by droplet RT-digital droplet PCR. Statistical analyses: Student’s t-test **p* < 0.05, n = 4–6. Data presented as mean ± SD.

Supplementary data 5 Ankle joint inflammation did not cause an increase in CD11b^+^ cells in the spinal cord parenchyma on day 14. A neglible number of CD11b^+^ cells were detected in the spinal cord parenchyma of uninduced and VEGFR2^ECKO^ mice and ankle joint CFA did not increase this number. 2-way ANOVA + Bonferroni’s multiple comparisons test, n = 3–6. Data presented as mean ± SD.
