# Supplementary figures and images for: VEGFR2 promotes central endothelial activation and the spread of pain in inflammatory arthritis
Source: Brain Behav Immun. 2018 Nov;74:49–67. doi: 10.1016/j.bbi.2018.03.012 (PMC6302073; doi:10.1016/j.bbi.2018.03.012)

**a****Day 11**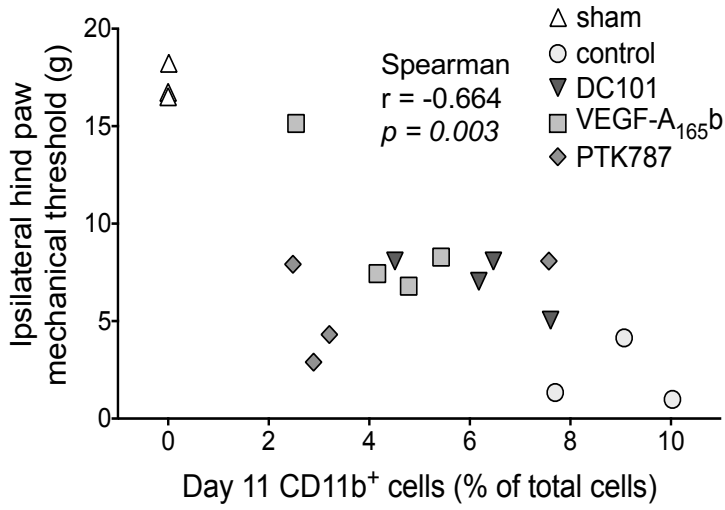**b****Day 8**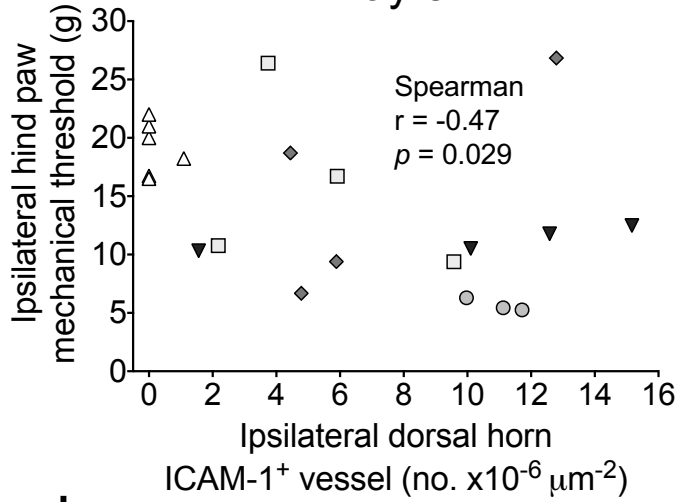**c****Day 11**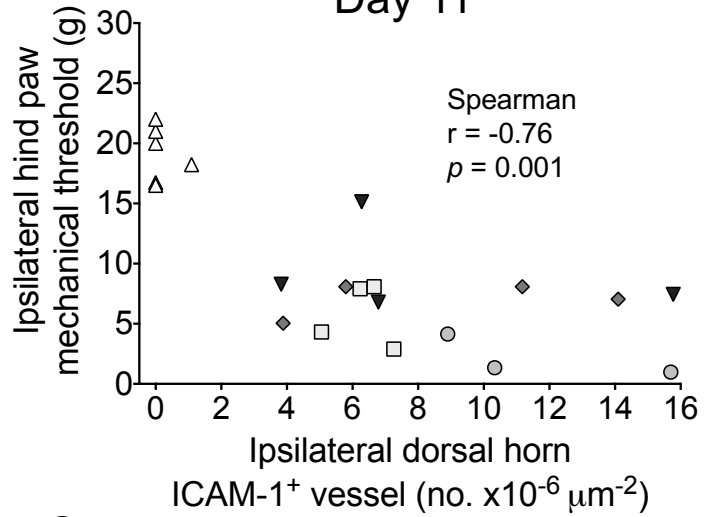**d****Day 8**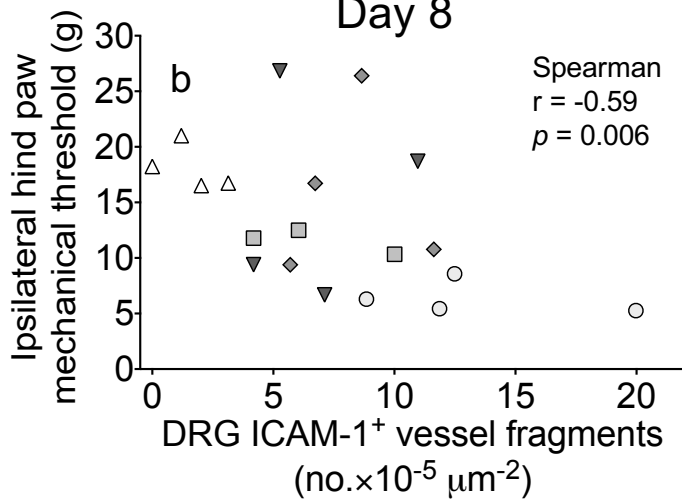**e****Day 11**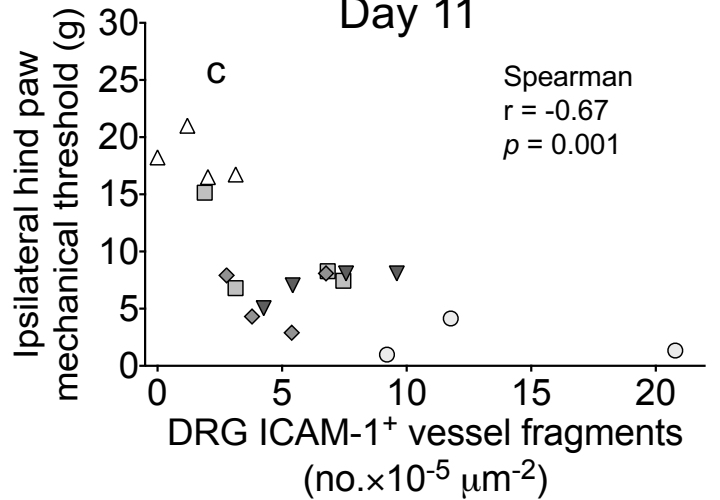

Supplement: Supplementary data 1 — Correlation analyses of CD11b+ dorsal horn parenchymal cell number and ICAM-1+ vessel number versus ipsilateral mechanical stimulus withdrawal threshold. The significant increase in dorsal horn CD11b+ cells induced by intra-articular CFA significantly and negatively correlated with the previous day’s reduced hind paw mechanical threshold (a). There was also a significant and negative association between dosal horn (b,c) and DRG (d,e) ICAM-1+ vessels and ipsilateral mechanical threshold at both time points. Statistical analyses: Spearman’s correlation test, n = 18–21. [file mmc1.pdf]

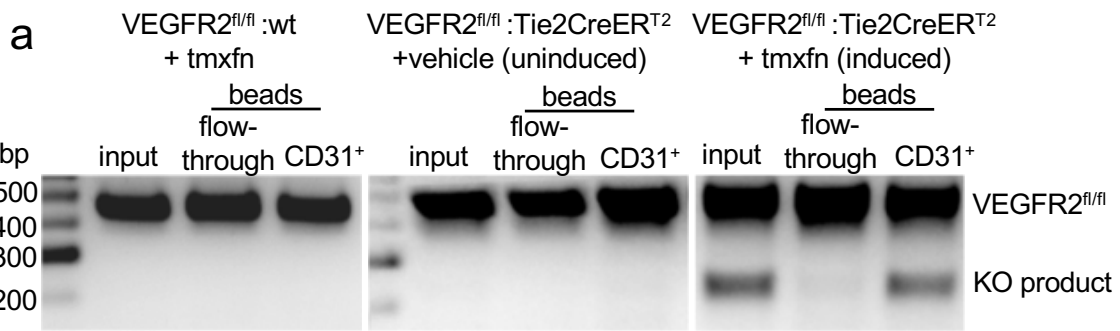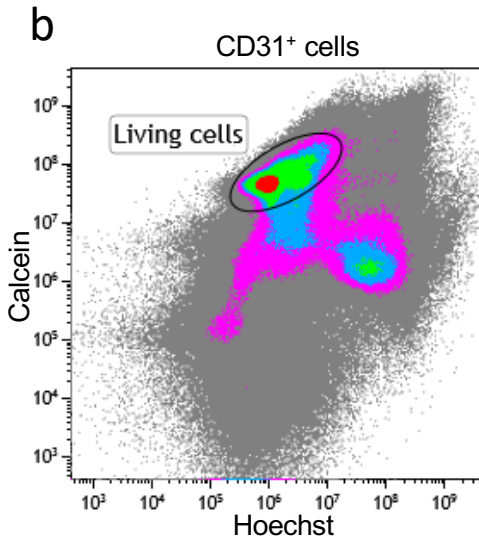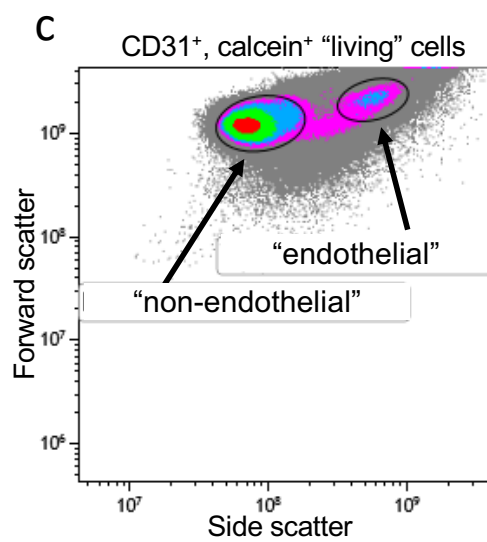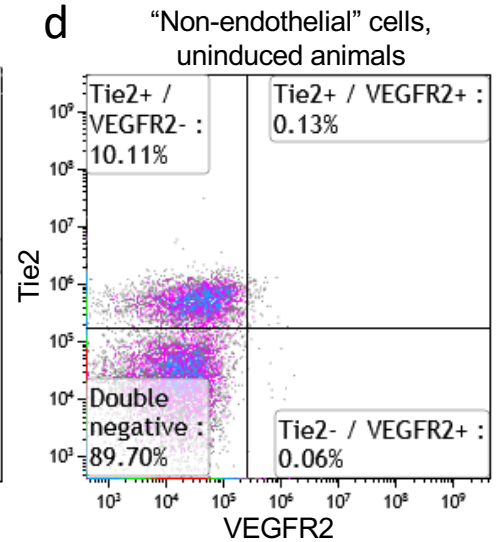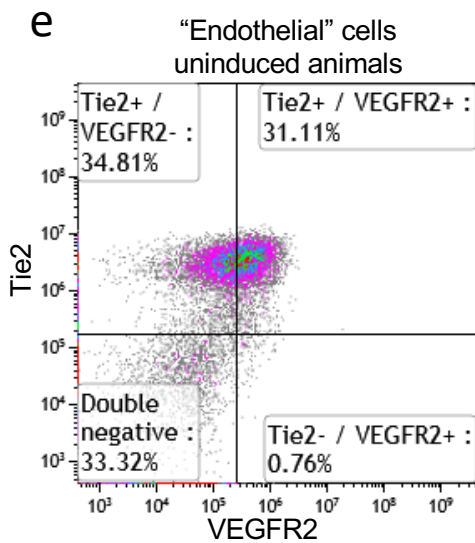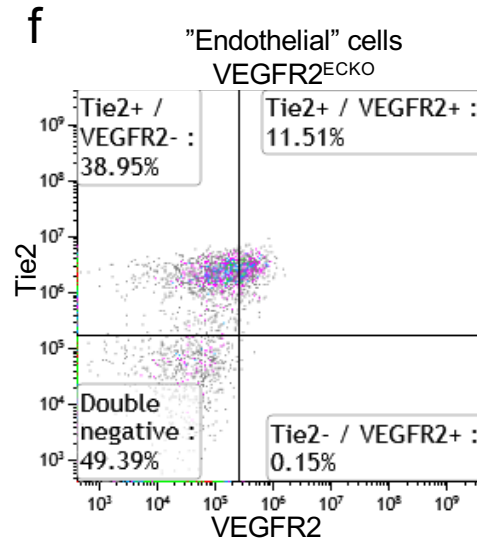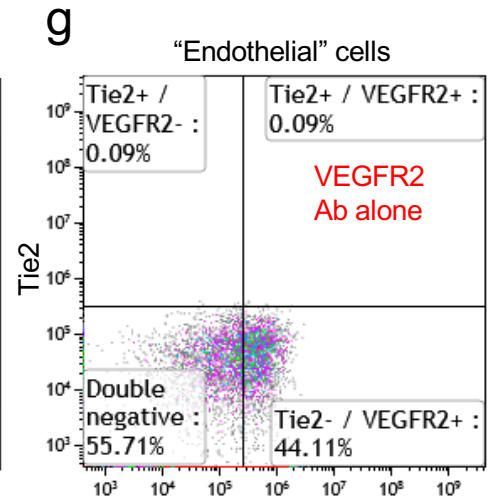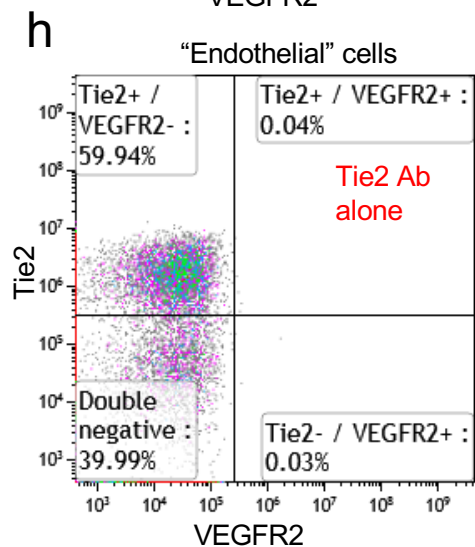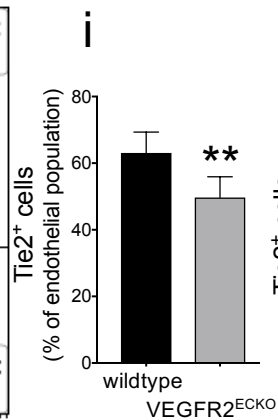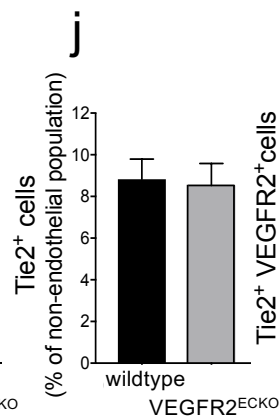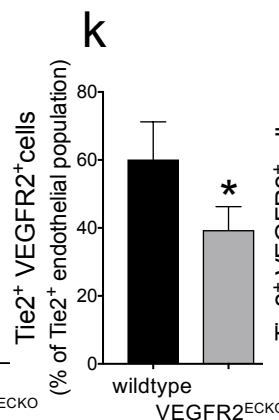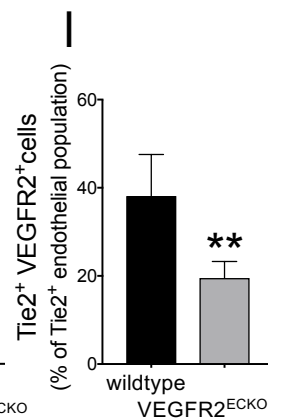

Supplement: Supplementary data 2 — Tamoxifen-induced Tie2CreERT2 VEGFR2 knock-out was investigated in primary CD31+ lung cells. The VEGFR2 knock-out gene product was detected only in CD31+ lung cells and not the flow-through fraction from tamoxifen-dosed VEGFR2fl/fl:Tie2CreERT2-positive mice indicating the VEGFR2 knock-out is inducible by tamoxifen and specific for CD31+ cells (n = 3) (a). Two distinct populations of living CD31+ cells (b; calcein+) were identified by scatter profile (c). A low Tie2/VEGFR2-negative (non-endothelial) population (d), and a high Tie2/VEGFR2-expressing (endothelial) population (e). An example of the endothelial population Tie2/VEGFR2 in KO mice (f). The number of Tie2+ cells in the endothelial population in VEGFR2ECKO and littermate control (d). Control stains using either Tie2 or VEGFR2 antibody alone revealed no channel compensation was required (g,h). Number of Tie2+ cells in the non-endothelial population (i). Percentage of Tie2+ cells that were VEGFR2+ in the endothelial population (j). Percentage of total endothelial population that were Tie2+/VEGFR2+ (k). VEGFR2 median fluorescence value of all Tie2+ cells within the endothelial population (l). Statistical analyses: student’s t-test: *p < 0.5, **p < 0.01. Data presented as mean± SD, n = 5–6. [file mmc2.pdf]

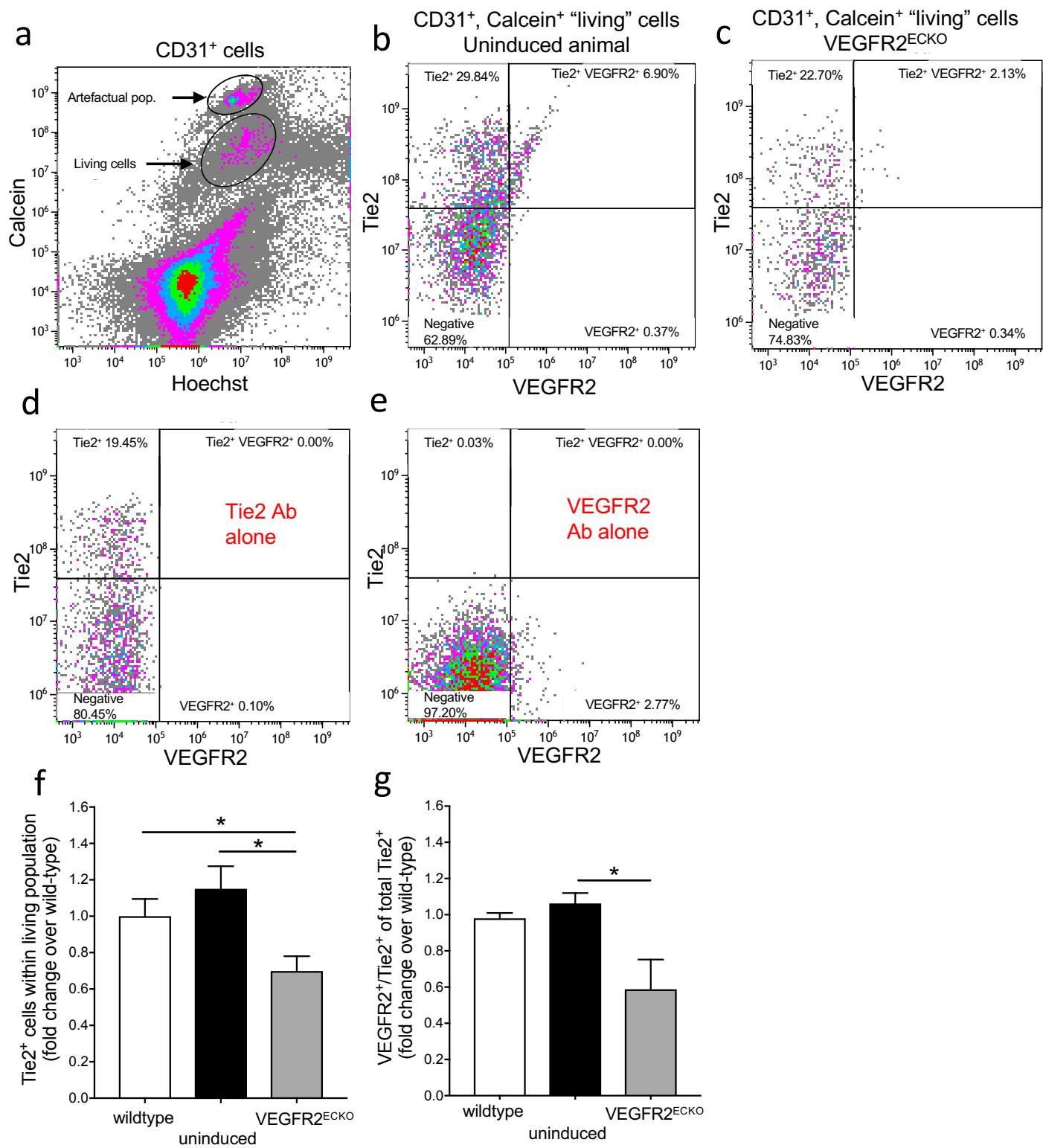

Supplement: Supplementary data 3 — Investigating the level of endothelial VEGFR2 knock-out by flow cytometry in the CD31+ spinal cord cells. No distinct populations of living spinal cord CD31+ cells (a; calcein+, Hoechst+) were identified by scatter profile (data not shown) so all living cells CD31+ were analysed. An artefactual population, possibly contaminating myelin, displayed properties not consistent with cells (a). An example of the Tie2/VEGFR2 in uninduced mice (b) and VEGFR2ECKO mice (c). Control stains using either Tie2 or VEGFR2 antibody alone revealed no channel compensation was required (d,e). Viable CD31+ Tie2+ cells as fold change of wildtype control (f) and VEGFR2+/Tie2+ of Tie2+ population as a fold change of wildtype control (g). Statistical analyses: 1-way ANOVA + Dunnett’s multiple comparisons test: vs. wildtype control, *p < 0.5, **p < 0.01, n = 5–8. Data presented as mean ± SD. [file mmc3.pdf]

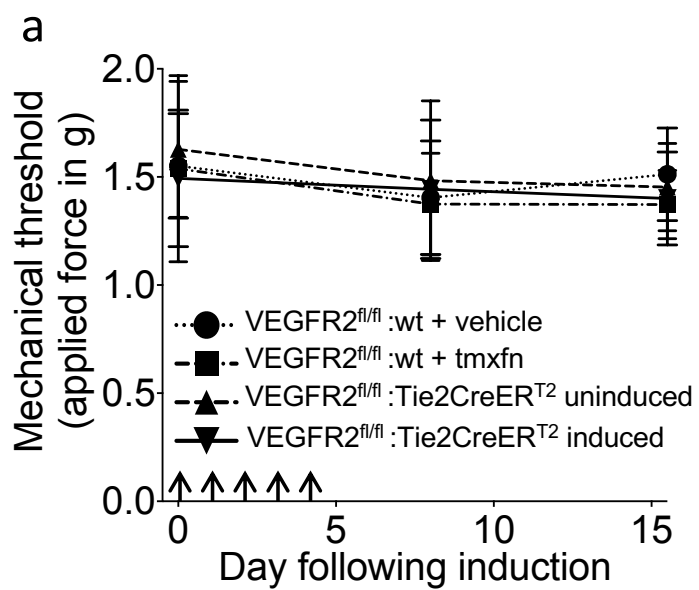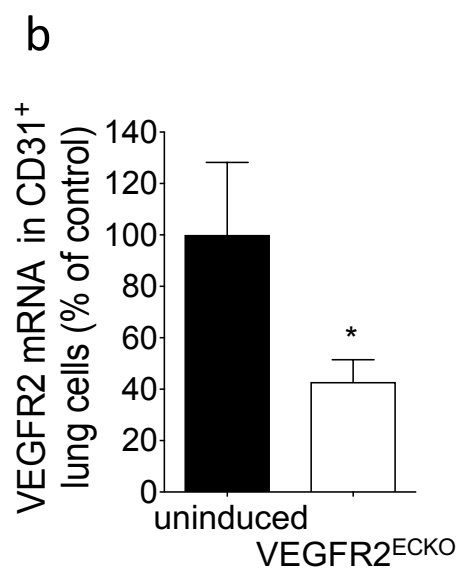

Supplement: Supplementary data 4 — VEGFR2ECKO did not affect mechanical threshold in uninflamed mice and caused a long lasting reduction in VEGFR2 mRNA in CD31+ lung cells. Treatment with tamoxifen or its vehicle had no effect on mechanical stimulus threshold in either VEGFR2ECKO, uninduced or wild type (wt) mice up to 2 weeks following the start of tamoxifen dosing (a). Following the completion of the ankle joint behavioral assessment (4 weeks after tamoxifen treatment) the level of VEGFR2 mRNA in CD31+ cells from knock-out mice was 57% lower compared with uninduced control indicating a long-lasting effect of the knock-out. Measured by droplet RT-digital droplet PCR. Statistical analyses: Student’s t-test *p < 0.05, n = 4–6. Data presented as mean ± SD. [file mmc4.pdf]

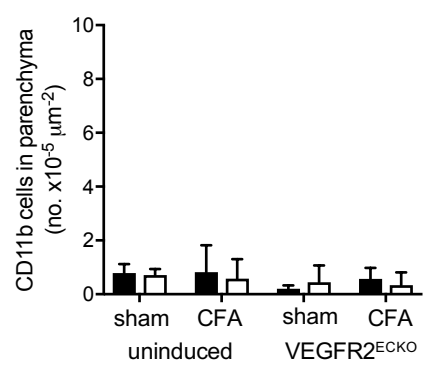

Supplement: Supplementary data 5 — Ankle joint inflammation did not cause an increase in CD11b+ cells in the spinal cord parenchyma on day 14. A neglible number of CD11b+ cells were detected in the spinal cord parenchyma of uninduced and VEGFR2ECKO mice and ankle joint CFA did not increase this number. 2-way ANOVA + Bonferroni’s multiple comparisons test, n = 3–6. Data presented as mean ± SD. [file mmc5.pdf]
